# Closed-Set Heterogeneous Domain Adaptation for IoT Intrusion Detection: An Anchor-Based Benchmark Across Single- and Multi-Source Transfer

**DOI:** 10.3390/s26113610

**Published:** 2026-06-05

**Authors:** Mohammad Chizari, Qublai Khan Ali Mirza, Abu Alam, Hassan Chizari

**Affiliations:** School of Business, Computing and Social Sciences, University of Gloucestershire, Park Campus, Cheltenham GL50 2RH, UK; qalimirza@glos.ac.uk (Q.K.A.M.); aalam@glos.ac.uk (A.A.); hchizari@glos.ac.uk (H.C.)

**Keywords:** IoT intrusion detection, heterogeneous domain adaptation, multi-source domain adaptation, semi-supervised learning, benchmark evaluation, GapClosure, representation contracts

## Abstract

**Highlights:**

**What are the main findings?**

- This paper introduces a controlled closed-set heterogeneous domain adaptation (HDA) benchmark for Internet of Things (IoT) intrusion detection with fixed contexts, representation contracts, anchor references, and twenty-seed paired statistical testing; at the 1:10 labelled-target ratio, GGA recovers 0.633–0.835 of the source-only-to-oracle headroom across C1–C4, while JSTN recovers 0.776–0.897 in the contemporary-source MS-HDA family and 0.872–0.926 in the mixed-vintage family.
- A same-budget comparison shows that DA’s advantage is not universal: some contexts favour adaptation, some show scarcity crossover, and some favour direct target-side supervised learning; PMGN and CWAN 1:10 method-coverage checks confirm that most deployment directions are not driven by a single selected method, and a compact ToN-IoT second-target confirmatory experiment shows the framework remains meaningful under a different IoT/IIoT target while confirming target-conditioned magnitudes.

**What are the implications of the main findings?**

- Closed-set HDA results should be interpreted against source-only, matched-budget target-only, and oracle target-only anchors, not as raw leaderboard scores.
- Native DA is deployment-valuable only when it recovers more operationally meaningful headroom than direct target-side labelling under the same resolved context and labelled-target budget.

**Abstract:**

Closed-set heterogeneous domain adaptation (HDA) for Internet of Things (IoT) intrusion detection aims to transfer detection capabilities across environments that differ in devices, telemetry, feature schemas, attack implementations, label taxonomies, and target supervision availability. Although recent HDA methods report strong performance, their deployment meaning is often unclear because improvements over a weak source-only baseline do not show how much target supervision headroom has been recovered or whether adaptation is preferable to direct target-side labelling under the same budget. This paper presents a controlled, anchor-based benchmark for closed-set HDA in IoT intrusion detection. Edge-IIoTset is used as the main fixed target dataset, with transfer from CICIDS2017, UNSW-NB15, CICIDS2017 + UNSW-NB15, and CICIDS2017 + NSL-KDD under single-source and multi-source settings. The benchmark defines fixed resolved contexts, Intersection and Union representation contracts, a five-class closed-set label contract, leakage-safe preprocessing, and an anchor ladder consisting of source-only, correlation alignment (CORAL), matched-budget target-only, and oracle target-only references. Geometric Graph Alignment (GGA) and the Joint Semantic Transfer Network (JSTN) are evaluated as the primary selected native single-source semi-supervised HDA (SS-HDA) and multi-source semi-supervised HDA (MS-HDA) exemplars, while the Prototype-Matching Graph Network (PMGN) and Conditional Weighting Adversarial Network (CWAN) provide 1:10 method coverage checks. Each method–context–ratio configuration is evaluated across twenty fixed seeds, and DA-versus-target-only differences are tested using paired seed-level statistical evidence. A compact second-target confirmatory experiment using ToN-IoT assesses whether the qualitative headroom recovery and same-budget deployment patterns remain visible under a different IoT/IIoT target. The results show that primary native HDA can recover substantial source-only-to-oracle headroom, but not uniformly. At the 1:10 labelled target ratio, GGA recovers 0.633–0.835 of the available headroom across C1–C4, while JSTN recovers 0.776–0.897 in the contemporary-source MS-HDA family and 0.872–0.926 in the mixed-vintage family. Same-budget comparisons show that DA is deployment-competitive only in some contexts; in others, direct target-side supervised learning is stronger. The benchmark therefore shows that closed-set HDA should be evaluated as target-conditioned, context-resolved evidence rather than as a pooled method leaderboard.

## 1. Introduction

When an Internet of Things (IoT) intrusion detection system (IDS) model trained in one environment is deployed in another, its realised performance depends on more than the adaptation algorithm. It also depends on which source and target domains are paired, which attack families are merged into a shared label space, which features are retained or materialised, how many target labels are allowed, and which reference baseline is used to judge success [1]. These decisions are especially consequential in heterogeneous IoT intrusion detection, where deployment environments may differ in device populations and telemetry interfaces [2,3]. They may also differ in feature schemas, collection pipelines, and attack implementations [4,5]. Without fixing these conditions explicitly, two studies can report similar macro-averaged F1 score (Macro-F1) improvements over source-only transfer while recovering very different fractions of the available target supervision headroom—a problem consistent with broader concerns about dataset-dependent IDS evaluation and unstable protocol design [6,7].

This problem matters because intrusion detection in IoT and Industrial Internet of Things (IIoT) environments is increasingly a transfer problem rather than a purely supervised classification problem. IoT and IIoT deployments now span critical and consumer-facing environments, including smart factories, connected healthcare, smart homes, and industrial control settings, increasing both operational value and exposure to attack [8,9]. Labelled target data are scarce and expensive to curate, while threat behaviour and deployment conditions continue to change [10,11].

This paper focuses on the closed-set semi-supervised part of this landscape. Consider a security team deploying an IoT detector in a new operational environment. This reflects a common IDS evaluation constraint: labelled attack traffic is expensive to obtain, difficult to keep representative, and sensitive to changes in collection environment [12,13]. However, a small labelled target subset may be feasible—for example, a few thousand flows curated during an initial deployment sprint, or a larger but still limited set curated over a longer operational period. At the same time, the team may have access to labelled source data from prior deployments or public benchmark corpora. Under this closed-set assumption, the source and target domains are mapped into a shared known label space, and the practical question becomes whether native HDA can convert source data, unlabelled target data, and limited labelled target data into useful target-domain detection.

Recent HDA methods have substantially expanded the algorithmic options for this setting, but the conditions under which their reported gains translate into deployment value remain underspecified [6,14]. Recent heterogeneous IDS benchmark work makes this concern more visible by showing that regime, dataset, and protocol choices can materially shape the interpretation of transfer results [15]. A raw improvement over source-only transfer is necessary but not sufficient. The improvement may be large in absolute Macro-F1 while still leaving most of the source-only-to-oracle target supervision gap unresolved. Conversely, a method may recover a high share of the available headroom but still be less attractive than simply training a target-only supervised model using the same labelled target budget. This paper therefore treats closed-set HDA evaluation as a supervision headroom problem, not only as a raw classification score comparison.

The benchmark developed in this paper evaluates native closed-set HDA under fixed and inspectable conditions. Geometric Graph Alignment (GGA) is used as the primary selected native single-source semi-supervised heterogeneous domain adaptation (SS-HDA) exemplar, while the Joint Semantic Transfer Network (JSTN) is used as the primary selected native multi-source semi-supervised heterogeneous domain adaptation (MS-HDA) exemplar [16,17]. To reduce the dependence on a single method choice, the benchmark also includes 1:10 method coverage checks using the Prototype-Matching Graph Network (PMGN) for single-source HDA and Conditional Weighting Adversarial Network (CWAN) for multi-source HDA [18,19].

The benchmark instantiates eight resolved closed-set contexts, where each context fixes the source–target tuple, label contract, representation contract, labelled target budget, and target test identity. Four SS-HDA contexts and four MS-HDA contexts are considered, each evaluated under both Intersection and Union representation contracts and at three labelled target ratios, 1:50, 1:10, and 1:5. The target test identity is fixed, preprocessing is leakage-safe, and comparisons are performed only within resolved contexts so that changes in tuple, representation contract, ratio, or supervision regime are not silently pooled. Here, native means that each method is evaluated only in the supervision regime that it is designed to support: GGA is used for SS-HDA, and JSTN is used for MS-HDA. The paper does not include adapter-based non-native portability rows.

The interpretive core of this paper is an anchor-based headroom analysis. Source-only transfer defines the naive cross-domain floor, while correlation alignment (CORAL) provides a lightweight classical alignment reference [20]. Matched-budget target-only learning asks what can be achieved by using the same labelled target subset directly for supervised target learning. Oracle target-only learning provides the full target supervision reference for the context family. These anchors allow raw Macro-F1 values to be converted into GapClosure, a normalised measure of how much of the source-only-to-oracle interval is recovered by native domain adaptation (DA). A same-budget comparison then asks a deployment-oriented question: whether native DA recovers more headroom than target-only supervised learning under the same labelled target budget.

The empirical findings show why this framing is necessary. Within the fixed Edge-IIoTset target setting, native SS-HDA with GGA recovers a large share of the available headroom in every SS-HDA context. At the 1:10 labelled target ratio, the primary SS-HDA exemplar, GGA, recovers a substantial but context-dependent share of the source-only-to-oracle headroom: GapClosure ranges from 0.633 in C4 to 0.835 in C1. The primary MS-HDA exemplar, JSTN, shows stronger headroom recovery under the evaluated multi-source conditions, but the results are interpreted by source-pair family rather than as a pooled leaderboard. In the contemporary-source family, C5–C6, 1:10 GapClosure ranges from 0.776 to 0.897; in the mixed-vintage family, C7–C8, it ranges from 0.872 to 0.926. These values justify the paper’s central claim: closed-set HDA can be highly effective, but its deployment value must be judged within the resolved context and against matched-budget target-only learning.

This paper focuses on the closed-set part of a broader auditable evaluation programme for heterogeneous IoT IDS domain adaptation. Unsupervised HDA, open-set DA, and benchmark-side portability adapters require different supervision contracts, output semantics, and evaluation metrics. They are therefore not mixed into the present empirical study. The narrower focus allows the paper to analyse closed-set SS-HDA and MS-HDA in greater deployment depth, especially through the comparison between native DA and matched-budget target-only learning.

This paper makes the following contributions.

1. **A controlled closed-set HDA benchmark for IoT intrusion detection.** The paper defines fixed resolved contexts covering SS-HDA and MS-HDA transfer into Edge-IIoTset, with declared source–target tuples, labelled target budgets, representation contracts, label contracts, fixed target test identities, and leakage-safe preprocessing.
2. **Anchor-based headroom interpretation with paired statistical evidence.** The paper introduces a deployment-oriented analysis based on GapClosure and a matched-budget target-only comparison, converting raw Macro-F1 improvements into claims about recovered supervision headroom and adaptation-versus-labelling trade-offs. Each DA-versus-target-only comparison is supported by paired seed-level statistical evidence, including bootstrap 95% confidence intervals, paired *t*-tests, Wilcoxon signed-rank checks, and effect size reporting.
3. **Native-regime empirical characterisation without pooled leaderboards.** The paper evaluates GGA and JSTN as primary selected native exemplars for SS-HDA and MS-HDA, respectively, rather than pooling methods into a single leaderboard. At the 1:10 labelled target ratio, GGA recovers 0.633–0.835 of the source-only-to-oracle headroom across C1–C4, while JSTN recovers 0.776–0.897 in the contemporary-source MS-HDA family and 0.872–0.926 in the mixed-vintage family.
4. **Method coverage and target-confirmatory evidence.** PMGN and CWAN method coverage checks at 1:10 test whether the main deployment patterns depend on a single method choice within each regime. A compact ToN-IoT second-target confirmatory experiment tests whether the qualitative headroom recovery framework remains visible under a different IoT/IIoT target.
5. **Reproducibility-oriented disclosure.** The paper reports split manifests, feature admissibility decisions, excluded feature lists, raw-label mappings, paired statistical-test outputs, and evidence bundle schema elements so that the reported scores can be traced back to their resolved evaluation contexts.

The study is organised around six research questions.

- **RQ1:** How does native SS-HDA behave under fixed source–target tuples, labelled target budgets, and representation contracts?
- **RQ2:** How does native MS-HDA behave under contemporary-source and mixed-vintage source-pair conditions?
- **RQ3a:** How much source-only-to-oracle target supervision headroom is recovered by native closed-set HDA within each resolved context?
- **RQ3b:** When compared at the same labelled target budget, does native DA recover more supervision headroom than direct target-only supervised learning?
- **RQ4:** Are the headline 1:10 deployment patterns sensitive to using only one selected method per regime, or are they supported by secondary PMGN and CWAN method coverage checks?
- **RQ5:** Do the qualitative headroom recovery patterns remain visible under a second IoT/IIoT target dataset, ToN-IoT, or are the observed magnitudes strongly target-conditioned?

**Figure 1:**
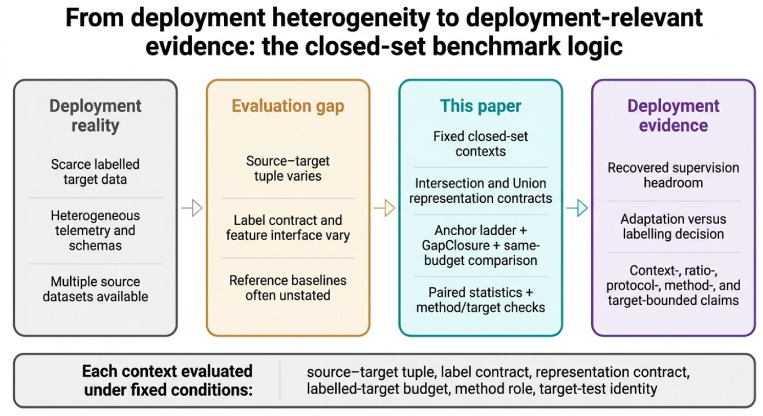
Evaluation logic for the closed-set HDA benchmark. Deployment heterogeneity in IoT intrusion detection creates confounded transfer claims through dataset choice, feature harmonisation, label contract, labelled target budget, method role, and reference baseline selection. The benchmark controls these factors through fixed closed-set contexts, paired Intersection/Union representation contracts, declared method roles, anchor references, GapClosure, matched-budget target-only comparison, paired seed-level statistical testing, and method coverage/second-target confirmatory checks. The resulting evidence is context-, ratio-, protocol-, method-, and target-conditioned, rather than a pooled leaderboard claim.

The overall evaluation logic that connects deployment heterogeneity, controlled benchmark contexts, anchor references, GapClosure analysis, and target-conditioned interpretation is summarised in Figure 1.

### Section Roadmap

The remainder of the paper is organised as follows. Section 2 reviews related work on closed-set HDA, heterogeneous IDS datasets, evaluation disciplines, and anchor-based DA interpretation. Section 3 defines the benchmark setup, including datasets, label contracts, representation contracts, contexts, methods, anchors, metrics, and admissibility checks. Section 4 reports the native closed-set DA results in raw Macro-F1 terms. Section 5 converts these results into supervision headroom recovery, compares native DA with matched-budget target-only learning, reports 1:10 method coverage checks for PMGN and CWAN, and presents the ToN-IoT second-target confirmatory experiment. Section 6 synthesises the deployment implications. Section 7 critically evaluates the benchmark scope and extension boundaries. Section 8 concludes the paper. The Appendices provide implementation details, split manifests, raw-label mappings, excluded feature lists, ToN-IoT second-target confirmatory disclosure, paired statistical test outputs, and evidence bundle information.

## 2. Related Work

Domain adaptation has become a major methodological response to the deployment problem in intrusion detection, where models trained in one collection environment often fail to retain useful discrimination in another. This problem is amplified in IoT and IIoT settings because the target environment may differ from the source in traffic distribution, device population, and protocol mix [21,22]. It may also differ in feature schema, label taxonomy, and data collection procedure, which makes direct cross-dataset comparison fragile [6]. This transfer framing is already visible in IDS-focused DA work, where source-domain supervision is reused to improve detection under target-domain shift [2,4].

This paper focuses on closed-set HDA because it represents a deployment-relevant case in which some labelled target data can be curated but full target supervision remains expensive. Under this assumption, the source and target domains are mapped into a known shared label space, and the same labelled target budget can be used either to support adaptation or to train a direct target-only supervised reference. This makes the comparison against matched-budget target-only learning meaningful, and it is central to the deployment interpretation developed in this paper. Unsupervised HDA and open-set DA address different deployment assumptions, especially the absence of labelled target data or the presence of unknown target classes [23,24]. These settings require different supervision contracts, output semantics, and evaluation metrics and are therefore treated as related but distinct from the closed-set benchmark studied here.

### 2.1. Closed-Set Heterogeneous Transfer for Intrusion Detection

Closed-set HDA methods for IDS attempt to exploit labelled source data and limited target information while respecting the fact that the source and target domains may expose different feature spaces. In IoT IDS, this setting is practically important because labelled IoT attack traffic is costly to obtain, while older or external IDS datasets may provide useful but imperfect supervision. The challenge is to transfer discriminative information without assuming that the source and target datasets share an identical representation.

GGA is the primary selected native exemplar for the SS-HDA setting [16]. It treats heterogeneous transfer as a representation alignment problem rather than assuming identical source and target feature spaces, and it was not designed specifically for Edge-IIoTset. What matters for this benchmark is that GGA provides a principled closed-set HDA mechanism that can be evaluated when one labelled source domain, a small labelled target subset, and an unlabelled target pool are available. In the present benchmark, GGA is therefore used to test whether native SS-HDA can recover supervision headroom under fixed source–target tuples, labelled target budgets, and representation contracts.

PMGN is included as a secondary single-source HDA method coverage check because it is explicitly designed for heterogeneous domain adaptation through prototype-level graph matching [18]. In this benchmark, PMGN is evaluated at the headline 1:10 labelled target ratio under the same closed-set label contract, representation contracts, target test identity, labelled target subset, and unlabelled target-training pool as the corresponding GGA contexts. It is reported separately from GGA so that the analysis can demonstrate whether the single-source headroom recovery and same-budget deployment patterns depend on one selected method.

JSTN is used as the primary selected native exemplar for the multi-source semi-supervised HDA setting [17]. Its semantic transfer and pseudo-label refinement design makes it suitable for testing whether multiple labelled sources provide complementary target-domain supervision rather than merely adding source-side volume. It addresses the case wherein more than one labelled source domain is available and attempts to use semantic transfer and pseudo-label refinement to bridge heterogeneous source and target spaces. This is directly relevant to IoT IDS deployment, where a practitioner may have access to multiple historical or external intrusion datasets but must adapt to a target IoT/IIoT environment. In this paper, JSTN is used as the primary selected native MS-HDA exemplar to test whether multi-source supervision improves closed-set transfer under contemporary-source and mixed-vintage source-pair conditions.

CWAN serves as the secondary multi-source HDA method coverage check. Its conditional weighting mechanism is relevant to the MS-HDA setting because source domains may differ in their conditional alignment with the target [19]. CWAN is evaluated at the headline 1:10 labelled target ratio under the same closed-set label contract, representation contracts, target test identity, labelled target subset, and unlabelled target-training pool as the corresponding JSTN contexts. Results are reported separately from those for JSTN so that the analysis can demonstrate whether the multi-source deployment pattern depends on one selected method.

Other DA approaches extend the broader heterogeneous IDS toolkit. Graph-based alignment methods model relational or structural correspondences across domains [16]. Adversarial and graph-convolutional approaches address unsupervised transfer when target labels are unavailable [23]. Open-set domain adaptation (OSDA) methods such as the Open-Set Domain Network (OSDN) address the further challenge that the target domain may contain attack families absent from the source label space [24]. Broader OSDA research similarly shows that unknown-class rejection cannot be reduced to ordinary closed-set prediction without changing the evaluation target [25]. These studies show that the field has developed strong regime-specific modelling approaches. The remaining benchmark question is how native closed-set methods behave under fixed contexts, explicit feature contracts, and declared target-label budgets, rather than only within locally defined experimental settings.

### 2.2. Datasets, Label Contracts, and Feature Heterogeneity

The empirical foundation for IDS transfer has expanded through widely used network, IoT, and IIoT datasets. CICIDS2017 and UNSW-NB15 provide widely used modern network intrusion detection system (NIDS) source domains with different collection settings and feature definitions [26,27]. NSL-KDD remains important as a legacy benchmark that still appears in transfer and robustness studies [7]. Edge-IIoTset and ToN-IoT provide IoT/IIoT-oriented target environments with different collection procedures, attack taxonomies, and telemetry assumptions [21,22]. In this paper, Edge-IIoTset is used as the main fixed target for the full benchmark, while ToN-IoT is used as a compact second-target confirmatory check (four contexts at the 1:10 labelled target ratio, defined in Section 5.3). These differences make such datasets valuable for evaluating transfer, but they also make uncontrolled comparisons difficult.

One central issue is label contract mismatch. Attack names that appear similar across datasets may not represent identical behaviours, while different raw attack families may need to be consolidated to support a stable closed-set task. Conversely, some raw labels may need to be excluded because they cannot be aligned credibly across the source and target domains. Without an explicit label contract, a reported performance difference may reflect a change in task definition rather than a change in adaptation capabilities.

A second issue is feature interface mismatch. Conventional NIDS datasets often expose flow-level statistical features, while IoT/IIoT datasets may include device-specific or protocol-specific telemetry. The same source–target tuple can therefore become a different empirical task depending on whether the evaluation retains only shared admissible features or allows broader feature union with materialised missing fields. This is why the representation choice should be treated as an experiment contract rather than a hidden preprocessing detail.

Benchmark resources that foreground heterogeneity are important because they make multi-regime and cross-domain IDS evaluation more explicit. TriHID is a recent example because it treats heterogeneous evaluation conditions as part of the benchmark design rather than as an incidental preprocessing issue [15]. The present paper builds on this direction but narrows the empirical focus to closed-set SS-HDA and MS-HDA so that the effects of the source tuple, label budget, representation contract, and matched-budget target-only learning can be analysed in greater depth.

### 2.3. Evaluation Discipline and Anchor-Based DA Interpretation

The broader cross-dataset IDS literature has shown that conclusions can shift substantially with dataset choices and preprocessing policies [6,14]. The train–test split design and evaluation context can also materially change the apparent strength of an intrusion detection model [5]. This is especially important for DA, where the target test set must remain isolated from training, preprocessing fit, hyperparameter selection, threshold tuning, and post hoc calibration. If these boundaries are not enforced, the transfer performance can be overstated through leakage or implicit target test adaptation.

DA benchmarking more generally has recognised the importance of contextual anchors. Source-only references define the naive transfer floor, while target-only references show what can be achieved when target supervision is available. Benchmarking work such as AdaTime uses source-only and target-supervised references to contextualise adaptation gains in time-series transfer [28]. Multi-source DA studies similarly use source-only and target-aware references to interpret whether adaptation is meaningful beyond ordinary supervised or naive transfer baselines [29]. These anchors prevent raw model scores from being interpreted without an empirical scale.

The present paper adapts this anchor-based logic to closed-set heterogeneous IoT IDS. Its contribution is not merely to report source-only and target-only baselines but to convert the source-only-to-oracle interval into a normalised supervision headroom scale through GapClosure. This is paired with a same-budget comparison against matched-budget target-only learning, which asks a deployment-facing question: given the same labelled target subset, is adaptation a better use of the available supervision than direct target-side supervised learning?

Within-context paired statistical evidence between adaptation and matched-budget target-only learning further supports anchor-based interpretation by quantifying whether the observed differences exceed seed-level variability under the same labelled target subsample.

### 2.4. Positioning of the Present Paper

Table 1 summarises how the present paper is positioned relative to the main strands of related work. The paper does not introduce a new DA algorithm. Instead, it provides a controlled closed-set benchmark and an interpretation procedure for native SS-HDA and MS-HDA in IoT IDS transfer.

The present paper applies this logic within a narrower closed-set scope. This separation allows the benchmark to focus on the practical closed-set question: when labelled target data are limited but legally available, how much headroom do native SS-HDA and MS-HDA recover, and when is adaptation preferable to direct target-only supervised learning under the same target-label budget?

The contribution is therefore evaluative. Native HDA methods are placed inside fixed closed-set contexts and interpreted against a declared anchor ladder, with deployment-oriented questions made empirically tractable. The benchmark combines GapClosure, matched-budget target-only comparison, Intersection/Union representation sensitivity, 1:10 PMGN/CWAN method coverage checks, twenty-seed paired statistical testing, and a ToN-IoT second-target confirmatory experiment. GGA and JSTN remain the primary closed-set semi-supervised native exemplars, while PMGN and CWAN provide method coverage checks under the same closed-set label contract, representation contracts, target test identity, labelled target subset, unlabelled target-training pool, and leakage boundaries. What the paper adds is the apparatus needed to translate raw Macro-F1 improvements into supervision headroom recovery, paired statistical evidence, target-conditioned interpretation, and a same-budget comparison against target-side supervised learning. These components are required for honest deployment claims in heterogeneous IoT IDS transfer.

## 3. Benchmark Setup

This section defines the closed-set benchmark used in this paper; therefore, it introduces only the framework components that directly support the claims made here: fixed evaluation contexts, declared representation contracts, explicit method role declarations, anchor-based interpretation, leakage-safe preprocessing, twenty-seed repeated-run reporting, paired statistical comparison, and second-target confirmatory evaluation.

The central design principle is that a reported score is not treated as a property of a method alone. It is treated as the outcome of a method executed under a resolved benchmark context. In this paper, a closed-set context fixes the source–target tuple, supervision regime, label contract, representation contract, split specification, labelled target budget, and target test identity. Comparisons are performed only within the same resolved context. A change from Intersection to Union, from 1:50 to 1:10, from SS-HDA to MS-HDA, or from one source–target tuple to another is therefore not a minor implementation variation; it defines a different benchmark condition.

The section is organised as follows. Section 3.1 defines the closed-set scope and maps the benchmark design to the paper’s research questions. Section 3.2 describes the datasets and derives the closed-set label contract. Section 3.3 defines the Intersection and Union representation contracts. Section 3.4 presents the eight Edge-IIoTset closed-set contexts and the compact ToN-IoT second-target confirmatory contexts. Section 3.5 declares the evaluated methods and their closed-set roles. Section 3.6 defines the anchor ladder and presents the GapClosure analysis. Section 3.7 specifies the metrics, aggregation, twenty-seed paired statistical testing, implementation disclosure, and admissibility checks.

### 3.1. Scope, Controls, and Research Question Mapping

The benchmark is restricted to closed-set HDA for IoT intrusion detection. It covers two supervision regimes: single-source semi-supervised HDA (SS-HDA) and multi-source semi-supervised HDA (MS-HDA). In both regimes, the source data are labelled, a small labelled subset of the target training pool is legally available, and the source and target domains are mapped into a shared benchmark label space. The target test set remains fixed and is never used for training, preprocessing fit, hyperparameter selection, model selection, threshold selection, or post hoc calibration.

Unsupervised HDA and open-set DA are not evaluated here because they require different supervision contracts, output semantics, and portability assumptions. The present paper therefore makes no claim about unknown class detection, unsupervised target adaptation, or benchmark-side adapter attribution. The empirical focus is closed-set transfer under limited labelled target data.

The closed-set scope allows the paper to answer six focused research questions.

- **RQ1:** How does the native SS-HDA method behave under fixed source–target tuples, labelled target budgets, and representation contracts?
- **RQ2:** How does the native MS-HDA method behave under contemporary-source and mixed-vintage multi-source transfer conditions?
- **RQ3a:** How much source-only-to-oracle target supervision headroom is recovered by native closed-set HDA across the reported regimes, ratios, and representation contracts?
- **RQ3b:** Under what conditions is native HDA preferable to matched-budget target-only learning, and when does direct target-side labelling dominate?
- **RQ4:** Are the headline 1:10 deployment patterns sensitive to using only one selected method per regime, or are they supported by secondary PMGN and CWAN method coverage checks?
- **RQ5:** Do the qualitative headroom recovery patterns remain visible under a second IoT/IIoT target dataset, ToN-IoT, or are the observed magnitudes strongly target-conditioned?

These questions require six benchmark controls. First, the target test identity must be fixed within each context family, so that score differences are not caused by resampling the evaluated target population. Second, the labelled target budget must be declared before execution, so that 1:50, 1:10, and 1:5 are not silently pooled. Third, the representation protocol must be explicit, because feature harmonisation changes the realised transfer problem. Fourth, method capability claims must be restricted to the declared role in which the method is evaluated. Accordingly, GGA is used as the primary selected native SS-HDA exemplar, while JSTN is used as the primary selected native MS-HDA exemplar. PMGN and CWAN are reported only as 1:10 method coverage checks and are not pooled with the primary method rows. Fifth, the paired DA-versus-target-only comparison must use the same labelled target rows within each seed. Sixth, target-conditioned interpretation must be explicit: Edge-IIoTset is the main benchmark target, while ToN-IoT is used only as a compact second-target confirmatory check.

**Figure 2:**
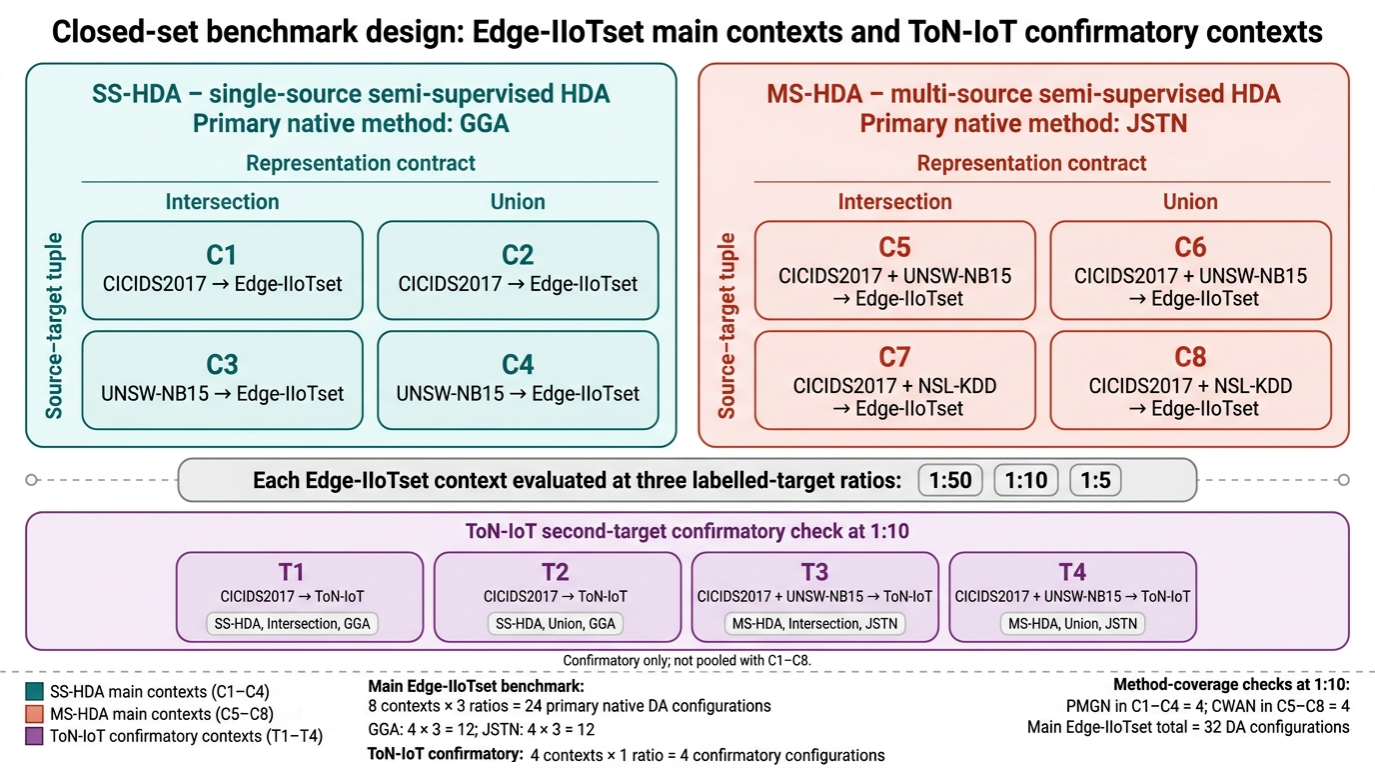
Closed-set benchmark design. Eight resolved Edge-IIoTset contexts are organised by supervision regime, source configuration, and representation contract. C1–C4 evaluate SS-HDA with GGA as the primary selected native exemplar, while C5–C8 evaluate MS-HDA with JSTN as the primary selected native exemplar. Each Edge-IIoTset context is evaluated at three labelled target ratios, 1:50, 1:10, and 1:5, giving 24 primary native DA method–context–ratio configurations. The benchmark additionally reports 1:10 method coverage checks using PMGN in C1–C4 and CWAN in C5–C8, adding eight secondary method–context configurations and yielding 32 main Edge-IIoTset DA configurations. A compact ToN-IoT second-target confirmatory experiment, T1–T4, is shown separately at the 1:10 ratio and is not pooled with C1–C8.

Figure 2 summarises this closed-set benchmark design by showing the eight Edge-IIoTset contexts, their supervision regimes, source configurations, representation contracts, labelled target ratios, secondary method coverage checks, and the separate ToN-IoT confirmatory setting.

### 3.2. Datasets and Closed-Set Label Contract Derivation

The benchmark uses Edge-IIoTset as the closed-set IoT/IIoT target dataset and evaluates transfer from four source configurations: CICIDS2017, UNSW-NB15, CICIDS2017 + UNSW-NB15, and CICIDS2017 + NSL-KDD. CICIDS2017 provides the principal modern source domain. UNSW-NB15 provides a second contemporary source family with different collection conditions, traffic composition, and feature definitions. CICIDS2017 + UNSW-NB15 provides a contemporary-source MS-HDA condition. CICIDS2017 + NSL-KDD provides a mixed-vintage MS-HDA stress condition in which a modern source and a legacy auxiliary source must be reconciled before transfer to the IoT/IIoT target [7,22,26,27].

In addition to the Edge-IIoTset main benchmark, the paper includes a compact second-target confirmatory experiment using ToN-IoT. This confirmatory experiment is not treated as a second full benchmark. It evaluates whether the qualitative headroom recovery and same-budget deployment patterns remain visible under a different IoT/IIoT target, using the headline 1:10 labelled target ratio, both representation contracts, the same twenty-seed policy, and the same anchor ladder. The ToN-IoT label mapping, split counts, feature-space summary, and excluded feature list are disclosed in Appendix E.

The benchmark treats each dataset as a processed benchmark variant rather than as a raw dataset name. Each variant is associated with a fixed admissible feature interface, a closed-set label mapping, a leakage-safe preprocessing policy, and a reproducible split specification. This matters because two studies can nominally use the same datasets while actually evaluating different tasks if they implement different feature exclusions, label merges, target test splits, or preprocessing decisions. Such variation is a known source of fragility in IDS evaluation and cross-dataset comparison [6,7].

The closed-set benchmark label space is fixed as:

(1) $$Y_{\mathrm{cs}}=\left\{\mathrm{normal},\;\mathrm{scan},\;\mathrm{DoS},\;\mathrm{DDoS},\;\mathrm{exploit}/\mathrm{credential}\;\mathrm{abuse}\right\}.$$

In this label contract, DoS denotes denial-of-service and DDoS denotes distributed denial-of-service. This five-class contract is a deliberate simplification. It is not intended to preserve the full attack taxonomy of every source dataset or of Edge-IIoTset. Instead, it creates a stable cross-dataset closed-set task with sufficient support across the selected tuples. Raw families that could not be aligned credibly across the source and target datasets are excluded from the closed-set contract rather than retained as sparse or semantically unstable classes.

Table 2 summarises the label contract resolution used in the benchmark. The key principle is that merges are performed only where the resulting benchmark class has a defensible shared meaning across the datasets. For example, different DoS subtypes are merged into the benchmark-level DoS class, while exploit-like and credential abuse behaviours are consolidated into a broader exploit/credential abuse class. Classes with insufficient or unstable cross-dataset alignment are excluded from the closed-set contract. This choice reduces the fine-grained taxonomic fidelity, but it prevents the benchmark from confusing class support mismatch with domain adaptation difficulty.

The consequence of this design choice is important. All closed-set Macro-F1, anchor, and GapClosure values reported in this paper are claims about performance on the five-class benchmark task in Equation (1). They are not directly comparable to studies that keep more Edge-IIoTset attack families, split DoS subtypes more finely, or define credential and exploit categories differently. This limitation is not a flaw in the benchmark; it is the condition that renders controlled cross-dataset comparison possible. The paper therefore prioritises a defensible, auditable closed-set contract over a broader but less stable taxonomy.

### 3.3. Representation Contracts: Intersection and Union

Heterogeneous IDS transfer is not only a distribution shift problem. It is also a representation problem: the source and target datasets may measure different fields, use different encodings, or expose different telemetry abstractions. Treating feature harmonisation as a preprocessing detail would therefore weaken the benchmark, because a method’s apparent performance could depend on an unstated choice about which features were retained, dropped, or materialised.

To make this choice explicit, every context is evaluated under one of two representation contracts. The Intersection contract retains only admissible features available across the relevant domains after cleaning and audit. It is the conservative comparison because it minimises missing feature materialisation and reduces the risk that performance differences are driven by artificial schema completion. The Union contract fixes an admissible feature superset before execution and materialises features absent from a particular domain using a deterministic benchmark rule. In this paper, features absent from a domain by schema are materialised before model fitting. Absent numeric features are materialised by zero-fill. A categorical feature absent from a domain’s schema is materialised as the ABSENT token for every row of that domain, while genuinely unseen categories encountered at transform time for a feature that exists in the schema are mapped to a separate unknown (UNK) token. All encoders are fit only on context-permitted training partitions.

The two contracts address different questions. Intersection asks how well a method transfers when representation confounds are minimised. Union asks how robust the method remains when schema mismatch is allowed to appear in the realised feature space. Union is therefore not treated as universally fairer or more realistic than Intersection. It is a controlled stress condition. A conclusion is strongest when it is directionally stable under both contracts; divergence between Intersection and Union is interpreted as substantive evidence of representation sensitivity rather than as random implementation noise.

Table 3 reports the realised closed-set feature-space summary. These counts are included in the main text because the representation contract is part of the empirical object being evaluated. Without them, “Intersection” and “Union” would remain abstract labels rather than inspectable benchmark conditions. The complete excluded feature lists corresponding to the exclusion counts in Table 3 are reported in Appendix D.

### 3.4. Closed-Set Evaluation Contexts

The benchmark contains eight resolved closed-set contexts, shown in Table 4. The design follows a balanced factorial logic: two SS-HDA source tuples are each evaluated under Intersection and Union, and two MS-HDA source-pair tuples are each evaluated under Intersection and Union. This produces four SS-HDA contexts, C1–C4, and four MS-HDA contexts, C5–C8.

CICIDS2017 → Edge-IIoTset is included as the principal modern-to-IoT transfer tuple. UNSW-NB15 → Edge-IIoTset is included to test whether the conclusions survive a different contemporary source family with a different feature interface and collection process. The MS-HDA design then separates contemporary-source diversity from mixed-vintage source divergence. CICIDS2017 + UNSW-NB15 → Edge-IIoTset represents a contemporary-source MS-HDA condition: both sources are modern NIDS datasets, but they differ in collection process, traffic composition, and feature interface. CICIDS2017 + NSL-KDD → Edge-IIoTset represents a mixed-vintage stress condition in which one modern source and one legacy auxiliary source must be reconciled before transfer to Edge-IIoTset.

This design is intentionally bounded rather than exhaustive. It does not test every possible source combination. Instead, it separates two questions that would otherwise be conflated: whether multi-source transfer benefits from contemporary source diversity, and whether it remains useful under legacy-source divergence. Reverse-direction transfer is not part of the main benchmark because the deployment scenario studied here is forward transfer from richer or pre-existing source datasets to a more constrained IoT/IIoT target. Reverse-direction transfer may be useful as a diagnostic extension, but it is not used to support the claims in this paper.

Each context is evaluated under three labelled target ratios:

(2) R={1:50,1:10,1:5}.

In this paper, 1:k means one labelled target-training instance for every *k* unlabelled target-training instances. The 1:50 ratio represents severe labelled target scarcity and tests whether a method can operate when only a very small target-label subset is available. The 1:10 ratio represents a moderate label budget and is used as the main interpretive middle point in the results. The 1:5 ratio represents a larger but still limited target-label setting. Its purpose is operational rather than statistical: it approximates the upper end of what a small security team might realistically curate by hand while still remaining within a semi-supervised transfer setting.

The Edge-IIoTset target-side split cardinalities for C1–C8 are reported in Table 5. The table makes explicit that the unlabelled target-training pool and target test set remain fixed, while the labelled target subset changes across ratios. The ToN-IoT confirmatory target uses a separate fixed split, reported in Appendix E.

Table 6 lists the four ToN-IoT second-target confirmatory contexts evaluated at the headline 1:10 labelled target ratio. These contexts mirror the SS-HDA/MS-HDA × Intersection/Union structure of C1–C8 but apply it to a different IoT/IIoT target, supporting RQ5.

### 3.5. Methods and Native Role Declarations

The benchmark distinguishes between primary selected native exemplars and secondary method coverage checks. The primary analysis uses GGA for SS-HDA and JSTN for MS-HDA because these methods match the two supervision regimes studied in the paper and can be executed under the declared closed-set label contract, representation contracts, leakage boundaries, and anchor ladder. To reduce the dependence on a single method choice within each regime, the benchmark adds one additional method coverage check per regime at the headline 1:10 labelled target ratio: PMGN for single-source HDA and CWAN for multi-source HDA.

Method inclusion follows five admissibility filters. First, the method must address heterogeneous source–target feature spaces rather than ordinary homogeneous adaptation. Second, it must match one of the evaluated supervision regimes without requiring unknown-class output semantics or target test labels. Third, its formulation must be sufficiently specified for benchmark-side implementation under leakage-safe preprocessing. Fourth, it must be executable under both Intersection and Union representation contracts without changing the benchmark label contract. Fifth, its role must be declared explicitly as either a primary selected native exemplar or a secondary method coverage check. The goal is not to create a pooled leaderboard of all HDA algorithms but to test whether the main headroom recovery and same-budget deployment patterns are stable beyond a single method choice within each regime.

For the secondary method coverage checks, PMGN and CWAN are executed under the same resolved closed-set contexts as the primary methods: the same source–target tuple, label contract, representation contract, target test identity, leakage-safe preprocessing, 1:10 labelled target subset, and unlabelled target-training pool are retained. Their results are reported separately as method coverage evidence rather than pooled with the primary method rows.

**GGA.** GGA is treated in this paper as the primary selected native SS-HDA exemplar. It is evaluated in the single-source contexts C1–C4 for primary SS-HDA capability claims. The method is used to test whether closed-set heterogeneous transfer can improve over source-only and CORAL when one labelled source domain and a small labelled target subset are available. Its output is a closed-set prediction over $Y_{\mathrm{cs}}$. No claim is made here that GGA is native to MS-HDA, and GGA is not used as primary MS-HDA evidence in this paper [16].

**PMGN.** PMGN is included as a secondary SS-HDA method coverage check at the 1:10 labelled target ratio. It is evaluated in C1–C4 under the same fixed source–target tuples, representation contracts, target test identity, and leakage-safe preprocessing used for GGA. Its role is not to redefine the primary SS-HDA benchmark but to test whether the SS-HDA headroom recovery pattern observed with GGA is also visible under a second heterogeneous adaptation method. The PMGN results are therefore reported separately from the primary GGA rows and they are not pooled into a single SS-HDA leaderboard [18].

**JSTN.** JSTN is treated in this paper as the primary selected native MS-HDA exemplar. It is evaluated in the multi-source contexts C5–C8, where Edge-IIoTset provides the target side, and the labelled source side is either CICIDS2017 + UNSW-NB15 or CICIDS2017 + NSL-KDD. Its benchmark role is to test multi-source semi-supervised heterogeneous transfer under contemporary-source diversity, mixed-vintage source divergence, and limited labelled target data. Like GGA, its output is a closed-set prediction over $Y_{\mathrm{cs}}$, but its primary capability claim is restricted to MS-HDA [17].

**CWAN.** CWAN is included as a secondary MS-HDA method coverage check at the 1:10 labelled target ratio. It is evaluated in C5–C8 under the same fixed source-pair tuples, representation contracts, target test identity, and leakage-safe preprocessing used for JSTN. Its role is to test whether the MS-HDA findings are specific to JSTN or whether similar headroom recovery and DA-versus-target-only patterns are observed under a second multi-source HDA method. The CWAN results are reported separately as method coverage evidence rather than pooled with those of JSTN [19].

Table 7 summarises the method roles used throughout the paper.

The main results and conclusions remain based on the primary selected native exemplars: GGA in C1–C4 and JSTN in C5–C8. PMGN and CWAN are used as 1:10 method coverage checks to test whether the headline findings depend on a single method choice within each regime. No adapter-based non-native portability rows are used to support the primary closed-set claims.

### 3.6. Anchors and the GapClosure Framing

The paper’s main interpretive contribution is not only that it reports whether a native HDA method improves the Macro-F1. It asks how much target supervision headroom is recovered by closed-set HDA. For this reason, every DA result is read against an anchor ladder rather than as an isolated score. The four anchors are shown in Table 8.

CORAL is included because it provides a simple but informative DA reference. It aligns the source feature covariance to the unlabelled target-training covariance using the standard second-order moment-matching formulation, without using target labels [20]. In this benchmark, CORAL is always fit under the same split, label contract, and representation protocol as the corresponding source-only and DA rows. It therefore tests whether a lightweight classical alignment step already closes meaningful headroom before more specialised HDA methods are credited with additional gains.

For MS-HDA contexts, the source-only and CORAL anchors are trained using the declared source pair for that context, with source partitions combined under the same preprocessing and representation contract used by the native MS-HDA method.

Ranking-based DA reporting can show that one method outperforms another, but it does not reveal whether the gain is practically meaningful. A method can outperform source-only transfer while still remaining far below target-native learning. Conversely, a method can approach the matched-budget target-only anchor, suggesting that adaptation is using limited target labels efficiently. The benchmark therefore converts the source-only-to-oracle interval into a normalised supervision headroom scale.

Let Φ(m,c,r) denote the Macro-F1 score of method *m* in context *c* at labelled target ratio *r*. Let $\Phi_{\mathrm{SO}}\left(c\right)$ denote the source-only Macro-F1 for context *c*, and let $\Phi_{\mathrm{TO}\text{-}\mathrm{oracle}}\left(c\right)$ denote the oracle target-only Macro-F1 for the same context family. GapClosure is defined as

(3) $$\mathrm{GapClosure}\left(m,c,r\right)=\frac{\Phi \left(m,c,r\right)-\Phi_{\mathrm{SO}}\left(c\right)}{\Phi_{\mathrm{TO}\text{-}\mathrm{oracle}}\left(c\right)-\Phi_{\mathrm{SO}}\left(c\right)}.$$

A GapClosure value of 0 means that the method has not improved over naive source-only transfer. A value of 1 means that the method has reached the oracle target-only reference. Values between 0 and 1 indicate partial recovery of the available supervision headroom. Negative values indicate that the method performs worse than source-only transfer. Values above 1 are possible and are not automatically invalid: in imbalanced closed-set tasks, a DA method may occasionally exceed the oracle target-only Macro-F1 on a particular seed or context because of rare-class recall behaviour or optimisation variance. Such cases are reported but inspected cautiously rather than treated as evidence that the oracle reference has been conceptually surpassed.

**Figure 3:**
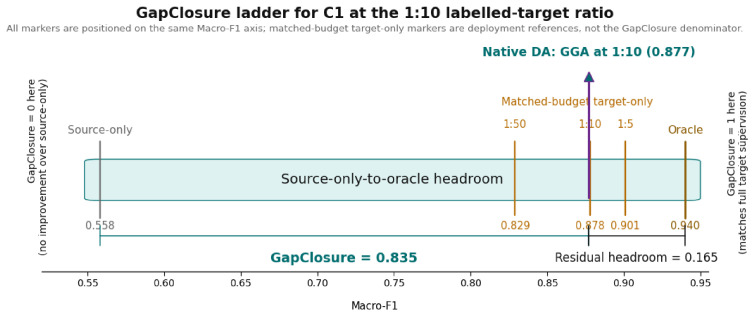
GapClosure ladder for context C1 at the 1:10 labelled target ratio. All markers are positioned on the same Macro-F1 axis between the source-only floor and the oracle target-only reference. Source-only reaches a Macro-F1 of 0.558, oracle target-only reaches 0.940, and GGA reaches 0.877, corresponding to GapClosure of 0.835 and residual headroom of 0.165. Matched-budget target-only markers are shown as deployment references at the same labelled target budget.

Figure 3 illustrates the GapClosure interpretation for context C1 at the 1:10 labelled target ratio, where the native GGA result lies almost exactly on the matched-budget 1:10 target-only reference and remains below the oracle target-only reference.

The oracle target-only anchor is used as the denominator because it is fixed within a context family and represents the full target supervision reference. The matched-budget target-only anchor is not used as the denominator because it changes with *r*. If the matched-budget anchor were used as the denominator, the GapClosure values would not be comparable across 1:50, 1:10, and 1:5. Instead, the matched-budget anchor is reported alongside each ratio as a practical decision reference: it shows whether DA is better than simply training on the same amount of labelled target data.

For example, consider C1 at the 1:10 ratio. With a source-only Macro-F1 of 0.558, an oracle target-only Macro-F1 of 0.940, and  native GGA reaching 0.877, GapClosure is

(4) $$\mathrm{GapClosure}=\frac{0.877-0.558}{0.940-0.558}=\frac{0.319}{0.382}\approx 0.835.$$

The interpretation is not that GGA solves the transfer problem completely. Rather, it recovers approximately 83.5% of the available source-only-to-oracle headroom in that context and ratio, while the remaining gap with oracle target-only shows that additional target supervision still has measurable value.

GapClosure involves within-context-family normalisation. It should not be read as an absolute measure of task difficulty across unrelated tuples. Two contexts can have the same GapClosure value but different absolute Macro-F1 gains if their source-only-to-oracle windows differ. For this reason, the results section reports both the raw Macro-F1 and GapClosure. The raw score shows the achieved performance level; GapClosure shows how much of the available supervision headroom has been recovered.

### 3.7. Metrics, Aggregation, Run Policy, and Admissibility

Macro-F1 is the primary metric because the closed-set IDS target task is class-imbalanced, and class-balanced performance is more informative than raw accuracy alone [30]. Accuracy and the Weighted-F1 are reported as secondary operational summaries. The area under the receiver operating characteristic curve (ROC-AUC) and area under the precision–recall curve (PR-AUC) are reported only where probabilistic outputs are semantically meaningful and stable across the relevant method outputs [31,32]. The main claims of the paper, however, are based on the Macro-F1 and GapClosure.

Aggregation is performed under the fixed-context rule. Each method–context–ratio configuration is repeated across twenty fixed random seeds:

(5) S={11, 17, 23, 31, 47, 53, 59, 67, 71, 79, 83, 89, 97, 101, 103, 107, 109, 113, 127, 131}.

The reported descriptive value is the mean ± standard deviation across these twenty runs. The same seed determines model initialisation and the labelled target subsample at each ratio. Therefore, within a seed, DA and matched-budget target-only learning use the same labelled target rows, ensuring that paired differences reflect the adaptation pipeline rather than different target-label draws.

The twenty-seed policy enables a paired statistical comparison between DA and matched-budget target-only learning. For each resolved context *c*, labelled target ratio *r*, and DA method *m*, paired seed-level differences in Macro-F1 are computed as

(6) $$d_{s}\left(m,\;c,\;r\right)=\Phi_{s}\left(m,\;c,\;r\right)-\Phi_{s}\left(\mathrm{matched}\text{-}\mathrm{TO},\;c,\;r\right).$$

where $\Phi_{s}$ denotes the Macro-F1 under seed *s*. For each comparison, we report the mean paired difference $\bar{d}$, a percentile bootstrap 95% confidence interval based on 10,000 resamples, a paired *t*-test, a Wilcoxon signed-rank robustness check, Cohen’s $d_{z}=\bar{d}/s_{d}$, and the matched-pairs rank-biserial correlation $r_{rb}$. Hypothesis tests use Holm–Bonferroni correction within three a priori inference families that answer distinct research questions: (i) the twenty-four primary GGA/JSTN comparisons across the eight Edge-IIoTset contexts and three labelled target ratios test the main deployment claim in RQ3a and RQ3b; (ii) the eight PMGN/CWAN 1:10 comparisons test method selection sensitivity in RQ4; and (iii) the four ToN-IoT 1:10 comparisons test target dependence in RQ5. Effect sizes and confidence intervals are treated as the main statistical evidence, while corrected hypothesis tests are interpreted as confirmatory checks of direction.

Family-level summaries are computed only for explicitly declared and coherent groups. GapClosure is a context-normalised quantity and is therefore interpreted first within each resolved context. Where descriptive summaries are reported, they are limited to declared regime family groups, such as C1–C4 for SS-HDA or C5–C6 and C7–C8 for the two MS-HDA source-pair families. The paper does not use pooled GapClosure means across heterogeneous source-pair families as leaderboard evidence. Intersection and Union are reported separately first and only then discussed as paired representation sensitivity evidence. SS-HDA and MS-HDA rows are interpreted according to their declared method roles: GGA and JSTN provide the primary method rows, while PMGN and CWAN provide 1:10 method coverage checks.

Leakage-safe preprocessing is enforced throughout the benchmark. Numeric coercion, non-finite handling, imputation, scaling, feature selection, and categorical encoders are fit only on context-permitted training partitions. The fixed target test set is excluded from all fitting, tuning, and model selection operations. Label handling is also fixed at the benchmark level and never reinterpreted per method. These restrictions are necessary because hidden leakage or method-specific label remapping would invalidate the interpretation of both the Macro-F1 and GapClosure.

A compact admissibility check is applied before aggregation. A run is retained only if (i) the executed split matches the declared context; (ii) the target test identity is unchanged; (iii) preprocessing is fit only on permitted training partitions; (iv) the closed-set label contract remains unchanged; (v) the representation protocol matches the declared context; (vi) the method role is correctly flagged; and (vii) the output record contains the required predictions, metrics, seed, and provenance fields. All results reported in this paper passed these admissibility checks.

The benchmark also retains labelled-set degeneracy checks for interpretation. These checks record per-class target-labelled support under each ratio, so that severe scarcity does not silently collapse a semi-supervised context into an effectively unsupported target task. They are used to support the analysis of label budget effects and to ensure that low-ratio settings remain valid closed-set semi-supervised contexts rather than accidental minority-class exclusion cases.

Implementation details are disclosed to support reproducibility. Neural DA methods are trained with Adam using an initial learning rate of $1\times 10^{-3}$, weight decay $1\times 10^{-4}$, mini-batch size 256, a maximum budget of 100 epochs, and early stopping patience of 15 epochs [33]. Model selection is based only on the context-permitted validation metric. The source-only, CORAL, matched-budget target-only, and oracle target-only anchor rows use the same tabular reference learner so that anchor differences reflect the training condition rather than a change in classifier family. In this benchmark, the reference learner is the Light Gradient Boosting Machine (LightGBM) configured with objective = multiclass, learning rate = 0.05, num_leaves = 31, feature_fraction = 0.8, bagging_fraction = 0.8, bagging_freq = 1, min_data_in_leaf = 20, maximum rounds = 500, and early stopping = 50 rounds [34]. The software stack, hardware manifest, split cardinalities, evidence bundle schema, raw-label mapping, excluded feature lists, ToN-IoT confirmatory disclosure, and paired statistical test outputs are reported in the Appendices; the corresponding machine-readable artefacts will be archived with the public repository accompanying the camera-ready version.

Table 9 summarises the repeated-run policy, seed list, method roles, optimiser settings, anchor learner, target-test restrictions, and admissibility requirements used in the closed-set benchmark.

## 4. Native Closed-Set DA Results

This section reports the native closed-set DA results under the benchmark setup defined in Section 3. The results are not presented as a pooled leaderboard. Instead, each method is interpreted only within its declared benchmark role. GGA is read as the primary selected SS-HDA exemplar in C1–C4, while JSTN is read as the primary selected MS-HDA exemplar in C5–C8. PMGN and CWAN are reported separately as 1:10 method coverage checks in Section 5.3 and are not pooled with the primary method rows. Source-only, CORAL, and oracle target-only anchors are reported alongside the native DA rows in the compact result tables. Matched-budget target-only references are summarised in the anchor overview and used explicitly in Section 5, where the DA-versus-labelling comparison is performed directly.

The Macro-F1 is the primary metric throughout this section. The mean and standard deviation are reported across the twenty fixed seeds defined in Equation (5). These summaries establish the descriptive raw score profile. Paired statistical comparisons between DA and matched-budget target-only learning are reported separately in Section 5 and the Appendix. GapClosure is not analysed in this section; it is treated separately in Section 5. The role of the present section is to establish the raw empirical performance profile from which the headroom analysis is derived.

### 4.1. Anchor Performance Across Closed-Set Contexts

The anchor results establish the interpretive scale for the native DA results. Source-only defines the naive cross-domain transfer floor. CORAL provides a lightweight second-order alignment reference under the same split, label contract, and representation protocol. Matched-budget target-only learning shows what can be achieved using the same labelled target budget available to the corresponding semi-supervised DA method. Oracle target-only learning provides the full target supervision reference for the context family.

Table 10 summarises the anchor Macro-F1 values across the eight closed-set contexts. Two patterns are expected to guide the later interpretation. First, source-only and CORAL remain below the target-only references in the already completed context families, confirming that the transfer tasks contain meaningful headroom for adaptation rather than only cosmetic gains over naive transfer. Second, the matched-budget target-only range increases with the labelled target budget, showing that the benchmark responds sensibly to additional target supervision. These anchors are therefore not auxiliary baselines; they define the performance window in which native closed-set HDA should be read.

The completed anchor rows already show why the anchor ladder is necessary. For example, C1 has a source-only Macro-F1 of 0.558 and CORAL has a Macro-F1 of 0.669, while oracle target-only reaches 0.940. A native DA score in this context should therefore be read not only as an improvement over source-only but also as a position within the larger source-only-to-oracle interval. The same logic applies across C1–C8: the anchor ladder defines whether adaptation is merely better than naive transfer, competitive with limited target-only learning, or still materially below target-native performance.

### 4.2. SS-HDA Results: GGA Across Single-Source Tuples

Table 11 reports the native SS-HDA results for GGA across C1–C4. These contexts evaluate closed-set transfer from one labelled source domain to Edge-IIoTset when only a limited labelled subset of the target training pool is available. The two source tuples separate the principal modern-source case, CICIDS2017 → Edge-IIoTset, from the more challenging contemporary-source alternative, UNSW-NB15 → Edge-IIoTset. Intersection and Union are reported separately because they define different realised feature spaces. These results primarily address RQ1 by showing how the primary SS-HDA exemplar behaves under fixed source–target tuples, labelled target budgets, and representation contracts.

Across C1–C4, GGA consistently improves over both source-only and CORAL. The improvement is largest in the more favourable CICIDS2017 → Edge-IIoTset tuple family and smaller in the UNSW-NB15 → Edge-IIoTset family, indicating that the second source tuple remains challenging even under the same target dataset, label contract, ratio schedule, and seed policy. This supports the benchmark design choice to include both SS-HDA tuples: the apparent value of SS-HDA is not independent of the source dataset used.

The ratio pattern is also informative. In all four SS-HDA contexts, GGA improves from 1:50 to 1:10, and the move from 1:10 to 1:5 produces a further but smaller gain. This suggests diminishing returns rather than complete saturation: a moderate labelled target budget provides the largest improvement over severe scarcity, while additional labelled target data continue to help but with a smaller marginal effect. This pattern motivates the GapClosure and DA-versus-target-labelling analysis in Section 5, where the native DA gains are compared directly against the results of matched-budget target-only learning.

### 4.3. MS-HDA Results: JSTN Across Multi-Source Tuples

Table 12 reports the native MS-HDA results for JSTN across C5–C8. These contexts evaluate whether multi-source semi-supervised heterogeneous transfer remains effective when the labelled source side contains either two contemporary but non-identical NIDS datasets or a mixed-vintage pairing of one modern source and one legacy auxiliary source. These results primarily address RQ2 by showing how the primary MS-HDA exemplar behaves under contemporary-source and mixed-vintage multi-source transfer conditions.

The contemporary-source MS-HDA tuple, CICIDS2017 + UNSW-NB15 → Edge-IIoTset, is reported as C5–C6. The mixed-vintage tuple, CICIDS2017 + NSL-KDD → Edge-IIoTset, is reported as C7–C8. This pairing allows the MS-HDA results to distinguish whether JSTN behaves differently under contemporary-source diversity and legacy-source divergence.

Across the MS-HDA contexts, JSTN provides a strong native multi-source result. It improves substantially over both source-only and CORAL in every context and at every labelled target ratio. At the 1:10 ratio, JSTN reaches 0.863 in C5 and 0.900 in C6 for the contemporary-source tuple, compared with source-only values of 0.561 and 0.551, respectively. In the mixed-vintage tuple, JSTN reaches 0.917 in C7 and 0.885 in C8, compared with source-only values of 0.518 and 0.500. Thus, the native MS-HDA method does not merely improve over naive transfer; it also exceeds the lightweight CORAL reference by a large margin across the reported multi-source contexts.

The mixed-vintage tuple is not weaker in the raw Macro-F1 results. Under Intersection, C7 is higher than C5 by 0.038–0.048 across ratios. Under Union, C8 is lower than C6 by 0.023–0.032. This pattern cautions against assuming that a contemporary-source pair is automatically easier than a modern–legacy source pair. Source-pair complementarity, the retained feature interface, class support, the representation protocol, and target mismatch all shape the realised transfer problem. The GapClosure analysis in Section 5 tests how much of the available source-only-to-oracle headroom these raw gains recover.

### 4.4. Representation Contract Sensitivity

The Intersection and Union protocols test whether the native closed-set conclusions depend on the feature harmonisation rule. Intersection reports the conservative shared-feature setting, while Union introduces materialised schema mismatch under the declared materialisation rule from Section 3.3. The comparison is therefore not a conventional hyperparameter robustness check; it is a test of whether the empirical conclusion survives a different representation contract. This analysis also supports RQ3a by showing that headroom recovery is conditioned by the realised representation contract rather than by the method name alone.

Table 13 reports the Union-minus-Intersection difference for each matched tuple, method, and ratio. Negative values indicate a lower Macro-F1 under Union, while positive values indicate a higher Macro-F1 under Union.

The representation contract effect is not uniform across regimes or source-pair families. For GGA in SS-HDA, Union produces a consistent penalty: a 0.049–0.064 Macro-F1 depending on the tuple and ratio. For JSTN in MS-HDA, the direction depends on the source-pair family. In the contemporary-source tuple, Union improves the Macro-F1 by 0.022–0.037, suggesting that the broader materialised feature interface provides a useful signal despite the schema completion burden. In the mixed-vintage tuple, Union reduces the Macro-F1 by 0.028–0.035, indicating that the additional materialised interface is less beneficial when the source pair already transfers strongly under Intersection. Representation sensitivity is therefore context-dependent rather than uniformly harmful.

**Figure 4:**
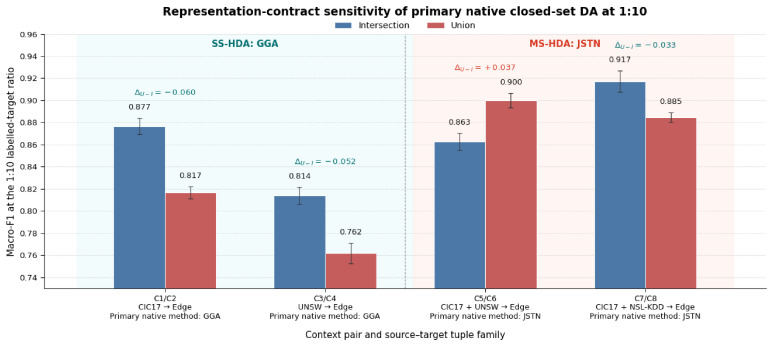
Representationcontract sensitivity of primary native closed-set DA at the 1:10 labelled target ratio. Bars report twenty-seed mean Macro-F1 under the Intersection and Union representation contracts for each matched source–target tuple family; error bars denote standard deviation. The annotation $\Delta_{U-I}$ denotes Union minus Intersection. The pattern is tuple-dependent: Union penalises the SS-HDA tuples, improves the contemporary-source MS-HDA tuple, and penalises the mixed-vintage MS-HDA tuple.

Figure 4 visualises this representation-contract sensitivity at the 1:10 labelled target ratio by comparing Intersection and Union Macro-F1 values across the SS-HDA tuples, the contemporary-source MS-HDA tuple, and the mixed-vintage MS-HDA tuple.

The contemporary-source MS-HDA tuple is the clearest example of this context dependence. Under Intersection, CICIDS2017 + UNSW-NB15 → Edge-IIoTset retains only 14 final features, the smallest realised interface in the benchmark. Under Union, the same source-pair family expands to 36 final features, including 22 Union-only materialised features. JSTN improves the Macro-F1 by 0.022–0.037 under this broader interface, suggesting that the Intersection contract is feature-starved for this tuple. This does not mean that Union is universally preferable: Union slightly penalises both SS-HDA tuples and the mixed-vintage MS-HDA tuple. Rather, the result shows that representation contracts can change the realised difficulty of a context in different directions depending on whether the limiting factor is schema mismatch or feature scarcity.

### 4.5. Label Budget and Seed Stability Profiles

The labelled target ratios reveal how each native regime responds to additional target supervision. Table 14 summarises the native Macro-F1 profile by ratio. For SS-HDA, the summary averages GGA across C1–C4. For MS-HDA, the summary averages JSTN across C5–C8, after separating contemporary-source and mixed-vintage behaviour in the preceding tables.

For GGA in SS-HDA, the largest improvement occurs between 1:50 and 1:10, followed by a smaller additional gain at 1:5. This pattern suggests diminishing returns: GGA benefits strongly from moving out of the most severe scarcity regime, but the marginal value of additional labelled target data decreases after the moderate 1:10 budget. The context-level range also shows that the tuple choice remains important: the UNSW-NB15 contexts remain more challenging than the CICIDS2017 contexts even under larger labelled target budgets.

For JSTN in MS-HDA, the raw Macro-F1 profile remains high in both source-pair families. In the contemporary-source family, the mean Macro-F1 increases from 0.858 at 1:50 to 0.881 at 1:10 and 0.894 at 1:5. In the mixed-vintage family, the corresponding values are 0.870, 0.901, and 0.907. The improvement with additional labelled target data is positive but modest, indicating that the primary MS-HDA method already extracts substantial transferable signal under severe scarcity. The seed-level standard deviations are comparable to those of GGA under the twenty-seed policy, suggesting stable optimisation behaviour under the reported settings.

Three findings emerge from the raw native result analysis and motivate the headroom analysis in Section 5. First, primary native closed-set DA improves over naive transfer in every context, with especially strong raw performance from JSTN in the MS-HDA contexts. Second, the label budget response differs by regime and source family: GGA in SS-HDA improves strongly from 1:50 to 1:10 and then gains more modestly at 1:5, whereas JSTN in MS-HDA starts from a high 1:50 baseline and continues to achieve steady but modest gains through 1:5. Third, the representation protocol interacts with the tuple and regime: Union penalises SS-HDA, improves the contemporary-source MS-HDA tuple, and penalises the mixed-vintage MS-HDA tuple. Section 5 therefore normalises the raw Macro-F1 results into a source-only-to-oracle supervision headroom scale and makes the adaptation-versus-labelling comparison explicit.

## 5. GapClosure and Supervision Headroom Analysis

Section 4 reported the native closed-set results in raw Macro-F1 terms. This section converts these results into supervision headroom recovery using the GapClosure measure defined in Equation (3). The purpose is to move beyond the question of whether a native DA method improves over source-only transfer and ask how much of the available source-only-to-oracle target-supervision window it recovers.

The analysis is regime-bounded. GGA is analysed as the primary selected SS-HDA exemplar in C1–C4, while JSTN is analysed as the primary selected MS-HDA exemplar in C5–C8. Because GapClosure is normalised by a context-specific source-only-to-oracle interval, it is interpreted primarily within a fixed context or a clearly declared context family. It should not be used as a pooled cross-context leaderboard or as an absolute measure of task difficulty across unrelated source–target tuples. The resulting GapClosure values should therefore not be read as a universal ranking between GGA and JSTN. They quantify how much headroom is recovered by the method assigned to each supervision regime under the fixed contexts, representation contracts, and labelled target ratios defined in Section 3.

### 5.1. Per-Context GapClosure

This subsection addresses RQ3a by quantifying how much source-only-to-oracle headroom is recovered within each resolved context. Table 15 reports GapClosure for each context and labelled target ratio. The values are computed using the source-only and oracle target-only anchors from Table 10. A value of 0 means that the native DA method performs no better than source-only transfer, while a value of 1 means that it reaches the oracle target-only reference for that context. Values between 0 and 1 indicate partial recovery of the available supervision headroom.

The per-context values show that both native regimes recover substantial source-only-to-oracle headroom, but with different profiles. In SS-HDA, GGA recovers a large share of the available headroom in every context. At the 1:10 ratio, GapClosure ranges from 0.633 in C4 to 0.835 in C1. This means that, even in the more challenging UNSW-NB15 → Edge-IIoTset contexts, native SS-HDA recovers more than half of the available supervision headroom.

The MS-HDA results show an even stronger headroom recovery pattern. JSTN achieves high GapClosure values across all four multi-source contexts. At the 1:10 ratio, GapClosure ranges from 0.776 in C5 to 0.926 in C7. Thus, the primary MS-HDA method does not merely improve over source-only transfer; under the reported source-pair conditions, it moves close to the oracle target-only reference in several contexts.

The budget pattern differs by regime and source family. In SS-HDA, the largest improvement generally occurs from 1:50 to 1:10, followed by a smaller additional gain at 1:5. This indicates diminishing returns rather than saturation or decline. In MS-HDA, JSTN also improves from 1:50 through 1:5, although the gains remain modest because the method already recovers a high share of the headroom at the lowest labelled target ratio.

This pattern is best interpreted as diminishing marginal headroom recovery rather than as evidence of a structural weakness in GGA. The GapClosure denominator, defined by the oracle target-only and source-only anchors, is fixed within each context and does not change with the labelled target ratio. As the labelled target budget increases, GGA has already recovered a large share of the available source-only-to-oracle interval, so the additional 1:10→1:5 gain is necessarily smaller in headroom terms. The observed pattern therefore indicates a reduced marginal benefit from additional labelled target data after moderate supervision, not a flaw in the native SS-HDA method.

**Figure 5:**
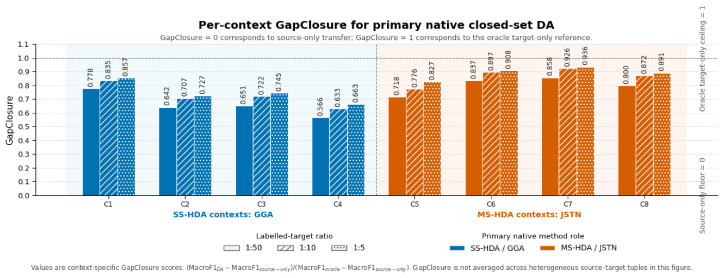
Per-context GapClosure for primary native closed-set DA across the eight Edge-IIoTset contexts. C1–C4 report GGA under SS-HDA, while C5–C8 report JSTN under MS-HDA. Bars show GapClosure at the 1:50, 1:10, and 1:5 labelled target ratios. GapClosure is normalised within each resolved context using the corresponding source-only and oracle target-only anchors; therefore, values indicate recovered source-only-to-oracle headroom within a context and should not be interpreted as a pooled cross-context leaderboard.

Figure 5 summarises this diminishing-return pattern by reporting per-context GapClosure across the 1:50, 1:10, and 1:5 labelled target ratios for the primary native SS-HDA and MS-HDA methods.

Although Figure 3 is drawn for C1 at the 1:10 labelled target ratio, the same ladder logic is used throughout the deployment synthesis: source-only, native DA, matched-budget target-only, and oracle target-only are interpreted as positions on the same Macro-F1 axis before judging whether adaptation recovers operationally meaningful headroom.

### 5.2. Regime Family GapClosure and Sign-Based Deployment Incidence

This subsection supports RQ3a and RQ3b by summarising headroom recovery within declared regime family groups and by reporting the sign-based deployment incidence without pooling heterogeneous source-pair families. Table 16 reports descriptive GapClosure summaries by supervision regime, source-pair family, and labelled target ratio. Because GapClosure is normalised by a context-specific source-only-to-oracle interval, the reported means are not used as pooled performance estimates across heterogeneous source–target tuples. They are retained only as within-family descriptive central tendencies for coherent context groups, and the accompanying spread indicates how much the context-level GapClosure values vary within the declared group. Cross-context deployment evidence is therefore reported separately using the sign-based same-budget comparison in Table 17.

Table 17 reports the same-budget deployment incidence within source tuple families rather than across pooled regimes. Each family contains two representation contract variants of the same source–target tuple, allowing the table to summarise the deployment direction without averaging the GapClosure magnitudes across heterogeneous tuples.

The regime family summary clarifies how the headroom evidence should be interpreted. Native SS-HDA with GGA recovers a large share of the available supervision headroom in every SS-HDA context. At the 1:10 ratio, the per-context GapClosure values range from 0.633 to 0.835, and, at 1:5, they increase to 0.663–0.857. In deployment terms, this means that, under the reported single-source contexts, a moderate labelled target budget combined with native SS-HDA recovers a substantial share of the source-only-to-oracle interval, but the same-budget comparison remains necessary.

Native MS-HDA with JSTN also recovers a large share of the available headroom, but the analysis reports this pattern by source-pair family rather than as a pooled C5–C8 estimate. Within the contemporary-source MS-HDA family, C5–C6, the descriptive mean GapClosure is 0.837 at 1:10 and 0.868 at 1:5. Within the mixed-vintage MS-HDA family, C7–C8, the corresponding values are 0.899 and 0.914. These summaries are interpreted as within-family representation contract sensitivity, not as cross-tuple performance estimates.

The internal MS-HDA split is also informative. Mixed-vintage MS-HDA recovers more headroom than contemporary-source MS-HDA at every labelled target ratio. At 1:10, the contemporary-source contexts C5–C6 recover 0.837 on average, while the mixed-vintage contexts C7–C8 recover 0.899. This confirms that the source-pair vintage alone does not determine MS-HDA difficulty. In this benchmark, the mixed-vintage tuple has a stronger raw Macro-F1 and stronger GapClosure, suggesting that source complementarity, retained feature interface, class support, and target alignment matter more than the dataset age alone.

The regime family result shows that both native regimes are effective, with JSTN in MS-HDA showing especially high headroom recovery under the reported source-pair conditions. The more important deployment question is how much of the remaining headroom is still worth pursuing through additional target-side labelling once native DA is already close to the oracle reference.

Several mechanisms may explain the strong MS-HDA recovery. First, the two-source setting provides broader class-discriminative evidence than a single-source transfer setting, and JSTN is designed to exploit joint semantic transfer across multiple labelled sources. Second, the auxiliary source may contribute complementary decision boundaries even when it is older or less deployment-realistic as a standalone source. This is especially visible in the mixed-vintage tuple, where CICIDS2017 + NSL-KDD achieves both a higher raw Macro-F1 and higher GapClosure than the contemporary-source tuple. Third, the smaller but more portable feature overlap in the mixed-vintage setting may be easier for JSTN to exploit than the more feature-starved contemporary-source Intersection interface. The benchmark does not isolate these mechanisms individually, but the combined effect is clear: under the reported closed-set contracts, native MS-HDA recovers most of the available source-only-to-oracle headroom.    

### 5.3. Same-Budget Comparison: DA Versus Matched-Budget Target-Only Learning

This subsection addresses RQ3b by comparing DA directly with matched-budget target-only learning under the same context, representation contract, labelled target ratio, seed, and labelled target subset. GapClosure measures recovery relative to the source-only and oracle target-only anchors, but deployment decisions also require a same-budget comparison. The matched-budget target-only anchor asks whether the labelled target subset available to DA would recover more headroom if used directly for target-side supervised learning. Table 18 reports this comparison at the same labelled target ratio. Positive values indicate that native DA recovers more headroom than matched-budget target-only learning; negative values indicate that direct target-side supervised learning recovers more.

This same-ratio comparison is stricter than comparing DA against source-only alone. It separates three deployment patterns: contexts where DA is stronger than target-only learning across the evaluated budgets, contexts where DA is competitive mainly under severe scarcity and is overtaken as the label budget grows, and contexts where matched-budget target-only learning dominates across all ratios.

The paired statistical layer supports the same-budget interpretation without replacing it. The mean paired ΔF1 and bootstrap confidence intervals quantify the practical margin between DA and matched-budget target-only learning under the same labelled target rows, while the paired *t*-test and Wilcoxon signed-rank test provide confirmatory direction checks after Holm–Bonferroni correction. Cases with a small $\Delta_{r}$, a small ΔF1, or confidence intervals crossing zero are interpreted as deployment ties rather than clear wins.

The same-ratio comparison shows that the deployment value of native DA is context-dependent. In SS-HDA, C1 is the clearest scarcity crossover case: GGA exceeds matched-budget target-only learning at 1:50, is approximately tied at 1:10, and is overtaken at 1:5. In C2 and C3, matched-budget target-only learning is stronger at all three ratios, although the difference is smaller than in C4. In C4, direct target-side supervised learning is clearly stronger across all evaluated budgets.

The MS-HDA comparison is mixed rather than uniformly favourable to either side. In C7, JSTN exceeds matched-budget target-only learning at 1:50 and 1:10 and is approximately tied at 1:5 under the $|\Delta_{r}|\leq 0.02$ tolerance. In C6, JSTN is stronger at 1:50, approximately tied at 1:10, and slightly below target-only learning at 1:5. In C5, matched-budget target-only learning is stronger at all three ratios. In C8, JSTN is approximately tied at 1:50 but falls below matched-budget target-only learning at 1:10 and 1:5. Thus, native MS-HDA can be deployment-competitive, but the advantage depends on the source-pair family, representation contract, and labelled target budget.

C7 at 1:5 illustrates the operational tolerance principle. Although the paired statistical test detects a stable direction because seed-level variability is small, the GapClosure difference is only +0.013, which falls within the $|\Delta_{r}|\leq 0.02$ operational tie tolerance. The deployment interpretation therefore prioritises the operational tolerance over isolated test significance and treats this case as approximately tied.

Table 19 reports selected paired DA-versus-matched-budget target-only comparisons, illustrating the main deployment patterns across primary Edge-IIoTset examples, method coverage checks, and ToN-IoT second-target confirmatory examples.

**Figure 6:**
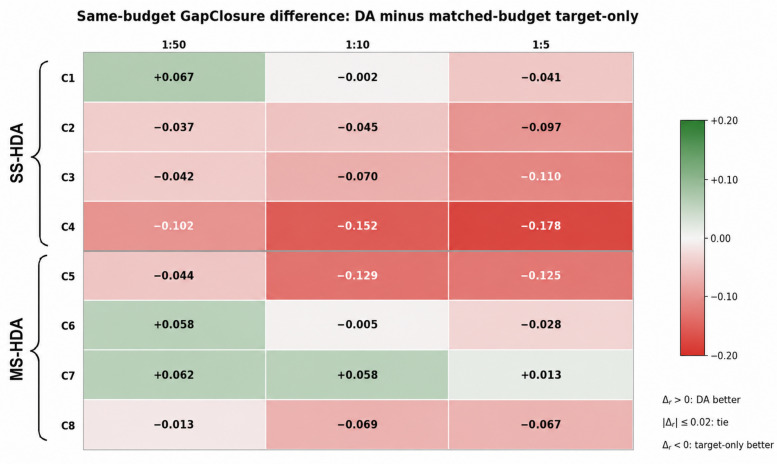
Same-budget GapClosure difference between primary native DA and matched-budget target-only learning across the eight closed-set Edge-IIoTset contexts. Each cell reports $\Delta_{r}={\mathrm{GapClosure}}_{DA}\left(c,r\right)-{\mathrm{GapClosure}}_{matched\text{-}TO}\left(c,r\right)$ for the same resolved context *c* and labelled target ratio *r*. Positive values indicate that DA recovers more source-only-to-oracle headroom than matched-budget target-only learning; negative values indicate that matched-budget target-only learning recovers more. Values with $|\Delta_{r}|\leq 0.02$ are operationally interpreted as approximate ties. The heatmap highlights scarcity crossover behaviour in C1 and C6, a persistent DA-favourable pattern in C7, and target-only-favourable patterns in C2–C5 and most of C8.

Figure 6 complements the selected paired statistical comparisons by visualising the same-budget GapClosure difference between primary native DA and matched-budget target-only learning across all eight Edge-IIoTset contexts and labelled target ratios.

The high upper end of the matched-budget target-only references remains important. In several contexts, matched-budget target-only learning at 1:5 approaches the oracle target-only reference. Given that the admissibility checks exclude target test leakage, the most plausible interpretation is target-only reference saturation on the five-class benchmark task: by the 1:5 budget, the labelled target subset is already large enough for the target-only learner to recover most of the oracle target-only performance. This finding is distinct from the diminishing marginal headroom recovery observed for DA. The deployment question is therefore not simply whether DA improves over source-only, but whether DA provides enough additional value over a strong matched-budget target-only baseline to justify adaptation engineering.

### 5.4. Method Coverage Checks at the 1:10 Labelled Target Ratio

This subsection addresses RQ4 by testing whether the headline 1:10 deployment directions depend on a single selected method per regime. The benchmark includes one secondary method coverage check for each supervision regime at the headline 1:10 labelled target ratio. PMGN is evaluated in the SS-HDA contexts C1–C4, while CWAN is evaluated in the MS-HDA contexts C5–C8. These checks use the same closed-set label contract, representation contracts, target test identity, labelled target subset, unlabelled target-training pool, leakage-safe preprocessing, and anchor ladder as the corresponding primary GGA and JSTN runs. The purpose is not to create a four-method leaderboard but to test whether the main headroom recovery and same-budget deployment patterns depend on a single method choice.

Agreement between the primary method and the secondary coverage method is assessed by deployment direction rather than by exact score equality. At 1:10, the two methods are treated as agreeing in a context if they fall on the same side of the matched-budget target-only reference: both exceed it, both are approximately tied with it, or both fall below it using the $|\Delta_{1:10}|\leq 0.02$ operational tolerance defined in Table 17. Divergence indicates method sensitivity within the same resolved context and is reported rather than averaged away.

The 1:10 method coverage GapClosure results are reported in Table 20, and their deployment-direction agreement with the corresponding primary methods is summarised in Table 21.

The method coverage checks show that the main 1:10 deployment patterns are not driven only by a single selected method. In SS-HDA, PMGN supports the GGA pattern across C1–C4: both methods are approximately tied with matched-budget target-only in C1 and fall below the matched-budget target-only reference in C2–C4, with PMGN showing slightly lower GapClosure than GGA in each context. In MS-HDA, CWAN mostly supports the JSTN pattern but reveals one method-sensitive case. Both methods remain below matched-budget target-only in C5, both support a DA-favourable direction in C7, and both remain below the strong target-only reference in C8. In C6, however, JSTN has an approximate tie with matched-budget target-only, while CWAN is slightly above it, indicating borderline method sensitivity under the Union representation contract. These results do not convert the benchmark into a pooled four-method leaderboard; instead, they identify where the deployment pattern is less method-dependent and where it remains method-sensitive within the same resolved context.

These method coverage results are used in Section 5.3 to distinguish deployment patterns that are less method-dependent from those that remain method-sensitive.

### 5.5. Second-Target Confirmatory Evidence Using ToN-IoT

This subsection addresses RQ5 by testing whether the qualitative headroom recovery and same-budget deployment patterns remain visible under a second IoT/IIoT target. The main benchmark deliberately fixes Edge-IIoTset as the target dataset so that the source tuple, representation contract, labelled target budget, and method role can be isolated. This fixed-target design strengthens the internal comparability but means that the absolute GapClosure values should be read as target-conditioned evidence. To assess whether the qualitative pattern is unique to Edge-IIoTset, the benchmark includes a compact ToN-IoT confirmatory experiment at the 1:10 labelled target ratio.

Table 22 reports the ToN-IoT second-target confirmatory results at the 1:10 labelled target ratio, showing whether the qualitative DA-versus-target-only deployment patterns remain visible beyond the main Edge-IIoTset target.

The ToN-IoT confirmatory results support the target-conditioned interpretation. The qualitative headroom recovery pattern remains visible under a second IoT/IIoT target: both SS-HDA and MS-HDA recover substantial source-only-to-oracle headroom, and the deployment direction still depends on the representation contract. Intersection favours DA in both ToN-IoT confirmatory tuples, while Union weakens or neutralises the DA advantage. These results do not convert the paper into a universal multi-target benchmark; instead, they show that the anchor-based interpretation is portable while absolute GapClosure magnitudes and same-budget deployment decisions remain target-dependent.

These confirmatory results are incorporated into the deployment synthesis in Section 5.3: they support the portability of the anchor-based interpretation while reinforcing that the absolute GapClosure magnitudes and same-budget decisions remain target-conditioned.

### 5.6. Residual Headroom and Deployment Decision Synthesis

This subsection synthesises RQ3b, RQ4, and RQ5 into deployment guidance by combining residual headroom, method coverage agreement, and second-target evidence. The preceding analyses provide three layers of deployment evidence: a primary GapClosure and same-budget comparison across C1–C8, method coverage checks using PMGN and CWAN at 1:10, and a second-target confirmatory check using ToN-IoT. Residual headroom, defined as 1−GapClosure, estimates how much of the source-only-to-oracle interval remains unrecovered by the evaluated DA method.

The residual analysis gives the clearest deployment interpretation. In SS-HDA, GGA leaves 0.276 residual headroom at the 1:10 ratio and 0.252 at 1:5. This supports a positive but bounded conclusion: under the reported single-source contexts, native SS-HDA recovers a large share of the available headroom, but additional target supervision still has measurable value. The residual shrinks from 1:10 to 1:5, but by a smaller margin than the gain from 1:50 to 1:10, indicating diminishing returns rather than complete saturation.

In MS-HDA, the residual headroom is generally smaller than in SS-HDA within the evaluated source-pair families. In the contemporary-source MS-HDA family, JSTN leaves residual headroom of 0.163 at 1:10 and 0.132 at 1:5. In the mixed-vintage MS-HDA family, the corresponding residual values are 0.101 and 0.086. These values are interpreted within their declared source-pair families rather than as a pooled C5–C8 residual estimate.

Table 23 summarises the remaining source-only-to-oracle residual headroom after primary native closed-set DA, grouped by SS-HDA and the two MS-HDA source-pair families.

#### Deployment Rule

Native DA is most valuable when it recovers operationally meaningful headroom that direct target-side supervised learning cannot recover under the same labelled target budget. The combined evidence supports three deployment patterns. First, some contexts favour DA or remain DA-competitive across the evaluated budgets, with C7 providing the clearest primary method case: JSTN exceeds matched-budget target-only learning at 1:50 and 1:10 and is operationally tied at 1:5. This DA-favourable direction is also supported by the CWAN method coverage check at 1:10. Second, some contexts show a scarcity crossover pattern, as in C1 and C6, where DA is strongest under severe labelled target scarcity but matched-budget target-only learning catches up or overtakes it as the labelled target budget grows. Third, some contexts favour direct target-side supervised learning, especially C4 and C5. The ToN-IoT confirmatory experiment shows that these decisions remain target-conditioned: Intersection favours DA in the confirmatory target, while Union weakens or neutralises the DA advantage.

Under the reported benchmark, GGA justifies SS-HDA engineering most clearly in the CICIDS2017 → Edge-IIoTset contexts and especially under severe labelled target scarcity. In the more challenging UNSW-NB15 → Edge-IIoTset contexts, the advantage over matched-budget target-only learning weakens, particularly under Union. For MS-HDA, JSTN recovers a high share of the source-only-to-oracle headroom in each C5–C8 context and is deployment-competitive in selected contexts, especially C7 and the low-budget C6 case. However, matched-budget target-only learning remains a strong reference and can approach oracle performance at larger labelled target budgets. The practical recommendation is therefore not simply to prefer DA or target-side labelling universally but to compare them under the same labelled target ratio, representation contract, and source-pair family.

The decision is therefore not whether DA is generally better than target-side labelling. It is whether a declared DA pipeline recovers more operationally meaningful headroom than matched-budget target-only learning under the same target, source tuple, representation contract, labelled target budget, and method role.

The main conclusion is not that closed-set DA recovers a single uniform share of target supervision headroom. The defensible conclusion is more precise: native SS-HDA with GGA recovers a large and stable share of the available headroom, while native MS-HDA with JSTN recovers an even larger share under the reported multi-source conditions. However, high DA GapClosure does not automatically mean that adaptation is the best deployment investment, because matched-budget target-only learning can also approach the oracle target-only reference at larger labelled target budgets. The central empirical finding is therefore twofold: native closed-set HDA can recover substantial supervision headroom in both single-source and multi-source settings, but its practical value must be judged against that of the matched-budget target-only anchor within each context, ratio, and representation contract.    

## 6. Synthesis and Deployment Implications

The closed-set benchmark supports a deployment-oriented interpretation rather than a single pooled method ranking. The main finding is not simply that native DA improves over source-only transfer. The stronger finding is that native closed-set HDA can recover a large share of the source-only-to-oracle supervision headroom in both SS-HDA and MS-HDA, but its practical value depends on the matched-budget target-only alternative within the same resolved context.

The 1:10 PMGN and CWAN results provide method coverage checks for the deployment synthesis. A deployment pattern is treated as less method-dependent when the primary method and the secondary coverage method fall on the same side of the matched-budget target-only reference in the same resolved context. When they diverge, the pattern is reported as method-sensitive rather than attributed to the whole SS-HDA or MS-HDA regime.

In the present benchmark, only C6 at 1:10 exhibits this divergence: JSTN is approximately tied with matched-budget target-only learning, while CWAN is slightly above it. This context is therefore treated as method-sensitive, whereas the remaining seven 1:10 contexts show agreement in deployment direction between the primary and coverage methods.

Across the main Edge-IIoTset benchmark, the primary native methods improve over source-only and CORAL in their declared regimes. GGA provides a stable primary SS-HDA reference across C1–C4, recovering 0.633–0.835 of the source-only-to-oracle headroom at the 1:10 labelled target ratio. JSTN provides a strong primary MS-HDA reference across the two declared source-pair families. At the 1:10 labelled target ratio, it recovers 0.776–0.897 of the source-only-to-oracle headroom in the contemporary-source family, C5–C6, and 0.872–0.926 in the mixed-vintage family, C7–C8. At 1:5, the corresponding ranges are 0.827–0.908 for the contemporary-source family and 0.891–0.936 for the mixed-vintage family. These results show that, under the reported source pairs and target conditions, the multi-source supervision signal supports particularly strong headroom recovery in this benchmark.

The same-budget comparison reveals three distinct deployment patterns. First, some contexts favour DA or remain DA-competitive across the evaluated budgets. C7 is the clearest example: JSTN exceeds matched-budget target-only learning at 1:50 and 1:10 and remains approximately tied at 1:5. Second, some contexts show a scarcity crossover. In C1 and C6, native DA is most competitive under severe labelled target scarcity, but matched-budget target-only learning catches up or overtakes it as the labelled target budget grows. Third, some contexts favour direct target-side supervised learning across all evaluated budgets, especially C4 and C5. C8 is more nuanced: it is approximately tied at 1:50, but target-only learning becomes stronger at 1:10 and 1:5. The practical implication is that the decision is not “DA or labelling” in general. It is whether the native DA pipeline recovers more headroom than matched-budget target-only learning under the same context, representation contract, and label budget.

This has direct deployment consequences for IoT IDS transfer. Native DA is most attractive when target labels are scarce, source data are reusable, and the adapted model recovers headroom that direct target-only learning cannot recover under the same budget. As the labelled target budget increases, the matched-budget target-only anchor becomes a stronger reference. In several contexts, target-only learning at 1:5 approaches the oracle target-only anchor, indicating target-only reference saturation on the five-class benchmark task. In such cases, the remaining value of adaptation engineering must be justified against a strong and simpler target-side supervised baseline, rather than only against source-only transfer.

The three deployment patterns can be read through the ladder logic illustrated in Figure 3. DA-favourable contexts place the DA marker above the matched-budget target-only marker; scarcity crossover contexts show this ordering changing as the labelled target budget increases; and target-only-favourable contexts place the matched-budget target-only marker above the DA marker across the evaluated budgets.

The representation contract results add a second deployment lesson. Intersection and Union are not interchangeable preprocessing options. In SS-HDA, Union slightly penalises GGA, suggesting that materialised schema mismatch can add noise. In MS-HDA, the effect depends on the source-pair family. Union improves JSTN for the contemporary-source tuple, where the Intersection interface is feature-starved, but reduces JSTN’s performance for the mixed-vintage tuple, where Intersection already transfers strongly. Representation design is therefore part of the deployment decision. A conservative shared-feature interface may be preferable when materialisation adds noise, while a broader Union interface may be beneficial when Intersection removes too much useful signal.

The ToN-IoT confirmatory experiment extends this deployment interpretation beyond the fixed Edge-IIoTset target. It shows that the anchor-based pattern remains meaningful under a second IoT/IIoT target: both SS-HDA and MS-HDA recover substantial source-only-to-oracle headroom, but the same-budget deployment direction still depends on the representation contract. In the ToN-IoT confirmatory contexts, Intersection favours DA, while Union weakens or neutralises the DA advantage. This supports the target-conditioned interpretation of the benchmark: the evaluation discipline is portable, but the absolute GapClosure magnitudes and deployment decisions remain target-dependent.

These findings argue against two common simplifications. The first is that one DA method can be ranked universally across heterogeneous IDS settings. The second is that improvement over source-only transfer is sufficient to justify deployment. The evidence supports a stricter interpretation: native DA should be evaluated within a resolved context, against an explicit anchor ladder, and against the matched-budget target-only alternative. Under this standard, closed-set HDA is often valuable, but its practical value is conditional, context-specific, and budget-dependent.

## 7. Critical Evaluation and Scope Boundaries

The benchmark is deliberately limited to closed-set heterogeneous domain adaptation. SS-HDA and MS-HDA are the supervision regimes evaluated in this paper; unsupervised HDA, open-set DA, unknown class detection, and adapter-based portability stress tests are not included. This restriction is a methodological choice rather than an oversight. These settings require different supervision contracts, output semantics, label assumptions, and evaluation metrics. Combining them with closed-set evaluation in one empirical paper would weaken the interpretability of the closed-set findings.

The five-class label contract is also a deliberate trade-off. The benchmark label space consists of normal, scan, DoS, DDoS, and exploit/credential abuse. This contract makes controlled cross-dataset comparison possible, but it necessarily reduces the taxonomic detail of the original datasets. The reported Macro-F1, GapClosure, and matched-budget comparisons are therefore conditioned on this five-class task. They should not be compared directly with studies that retain more Edge-IIoTset attack families, split DoS subtypes more finely, or define exploit and credential abuse behaviours differently. The advantage of the present design is auditability and comparability; the cost is reduced attack taxonomy granularity.

The dataset and method choices are illustrative rather than exhaustive. The main benchmark uses Edge-IIoTset as the fixed target dataset and evaluates transfer from CICIDS2017, UNSW-NB15, CICIDS2017 + UNSW-NB15, and CICIDS2017 + NSL-KDD. These source configurations separate single-source transfer, contemporary-source multi-source transfer, and mixed-vintage multi-source transfer. ToN-IoT is included as a compact second-target confirmatory check rather than as a second full benchmark. Similarly, GGA and JSTN are used as primary selected native exemplars, while PMGN and CWAN provide 1:10 method coverage checks. Other source datasets, target datasets, and native HDA algorithms may produce different absolute scores or different same-budget crossover patterns. The contribution of this paper is therefore not a final leaderboard but a fixed benchmark structure and an interpretation method for closed-set HDA performance.

The fixed-target design is also a scope boundary. In the main benchmark, Edge-IIoTset is held fixed as the closed-set target so that variations in performance can be attributed to the source configuration, representation contract, labelled target ratio, and native method role rather than to a changing target environment. This control is useful for evaluating source-side and representation-side effects, but it does not fully represent dynamic IoT deployments in which the target environment may itself change over time because of new devices, altered traffic patterns, updated firmware, emerging attack implementations, or changes in data collection infrastructure.

The same evaluation discipline could be extended to heterogeneous target adaptation by treating each target environment, target site, or target time window as a separate resolved target context. In such a design, the benchmark would need to declare a target-specific label contract, representation contract, target-training pool, fixed target test identity, and anchor ladder for each target condition. Multi-target or time-evolving target adaptation would therefore be handled analogously to the multi-source design used in this paper, but with the heterogeneity placed on the target side rather than only on the source side. Under this extension, GapClosure and matched-budget target-only comparison would remain useful, but they would have to be interpreted within each target context before any cross-target synthesis is attempted.

The representation contracts are controlled approximations of a larger feature harmonisation design space. Intersection minimises schema materialisation by retaining shared admissible features, while Union materialises absent features using deterministic benchmark rules. These two contracts make the representation choice explicit, but they do not exhaust all possible harmonisation strategies. Learned feature translation, semantic feature grouping, representation distillation, causal feature selection, or expert-engineered mappings may produce different outcomes. The results should therefore be read as evidence under two declared and reproducible representation contracts, not as proof that Intersection and Union are the only valid ways to handle heterogeneous IDS feature spaces.

The anchor ladder is a strength of the benchmark, but it also defines the boundaries of the interpretation. Source-only, CORAL, matched-budget target-only, and oracle target-only provide an interpretable performance scale. GapClosure is normalised relative to these anchors. This is useful because it converts the raw Macro-F1 into the recovered supervision headroom, but it also means that GapClosure is not an absolute method property that is independent of the benchmark design. A different anchor learner, a stronger target-only classifier, or a different oracle training convention could change the source-only-to-oracle window and therefore the resulting GapClosure values. This is not a defect of the measure; it is the reason that the anchor ladder must be declared and reported.

A noteworthy property of the benchmark is that matched-budget target-only learning approaches oracle target-only performance at the 1:5 ratio in several contexts. This is consistent with target-only reference saturation on the five-class task at moderate label budgets. It is itself an empirical finding: for this level of class granularity, 20,000 labelled Edge-IIoTset target instances may already capture most of the target-supervised performance available to the reference learner. Whether this saturation persists under finer attack taxonomies, noisier labels, smaller minority-class support, additional target domains, or larger and more diverse IoT/IIoT deployments remains an open question.

The statistical reporting layer combines descriptive and paired confirmatory evidence. Each method–context–ratio configuration is repeated across twenty fixed seeds and reported as the mean ± standard deviation in the main result tables. Paired seed-level differences between DA and matched-budget target-only learning are computed using the same seed and labelled target subset within each context and are reported with bootstrap 95% confidence intervals, paired *t*-tests, Wilcoxon signed-rank checks, Cohen’s $d_{z}$, and matched-pairs rank-biserial effect sizes. Holm–Bonferroni correction is applied within the declared inference families: the primary GGA/JSTN comparisons, the PMGN/CWAN method coverage comparisons, and the ToN-IoT confirmatory comparisons. Statistical significance is not interpreted as deployment value by itself; it is read together with GapClosure, the effect size, the same-budget deployment direction, and the residual headroom. The unit of interpretation remains the resolved context rather than a random sample from all possible DA tasks.

The deployment guidance is empirical rather than operational. The same-budget comparison treats labelled target instances as a uniform resource, but real deployment decisions also depend on the annotation cost, analyst time, data-sharing constraints, compute cost, update frequency, model governance requirements, and the operational risk of maintaining an adaptation pipeline. The benchmark provides evidence about performance trade-offs under controlled conditions. It does not replace site-specific cost modelling or security-risk assessment.

The method selection process is not a claim that GGA, PMGN, JSTN, or CWAN exhaust the closed-set HDA design space. GGA and JSTN are used as primary selected native exemplars for the two semi-supervised regimes, while PMGN and CWAN provide 1:10 method coverage checks under the same closed-set contexts. The benchmark therefore reports evidence about the evaluated methods under declared contexts and not about all possible SS-HDA or MS-HDA algorithms. The protocol itself is method-agnostic: additional native HDA methods can be added by declaring their supervision contract, target-label use, representation compatibility, leakage boundaries, and anchor comparisons before execution.

The ToN-IoT experiment should be read as a confirmatory target sensitivity check, not as an exhaustive multi-target benchmark. It tests whether the qualitative headroom recovery logic remains visible under a second IoT/IIoT target, but it is not sufficient to estimate universal cross-target performance. Future work should extend the same resolved-context discipline to additional targets, time windows, device families, and attack taxonomy variants.

Finally, the present article remains limited to closed-set semi-supervised HDA. Broader extensions to unsupervised, open-set, and adapter-based portability settings would require additional supervision contracts and output semantics and are therefore treated as future extensions rather than mixed into the present benchmark. The findings should therefore be read as claims about controlled closed-set HDA for IoT IDS, not as claims about every form of domain adaptation for intrusion detection.

## 8. Conclusions

This paper presented a controlled closed-set heterogeneous domain adaptation (HDA) benchmark for IoT intrusion detection. The benchmark was designed around fixed resolved contexts, explicit representation contracts, declared method roles, leakage-safe preprocessing, anchor-based interpretation, twenty-seed repeated-run reporting, a paired statistical comparison, method coverage checks, and second-target confirmatory evaluation. Rather than treating a score as a property of a method alone, the paper treats each result as the outcome of a method executed under a declared source–target tuple, supervision regime, label contract, representation contract, labelled target budget, and fixed target test identity.

The empirical results show that primary native closed-set HDA can recover substantial source-only-to-oracle target supervision headroom. In SS-HDA, GGA consistently improves over source-only and CORAL across C1–C4 and recovers between 0.633 and 0.835 of the available headroom at the 1:10 labelled target ratio, increasing to 0.663–0.857 at 1:5. In MS-HDA, JSTN also provides strong recovery, with GapClosure ranging within 0.776–0.897 in the contemporary-source family and 0.872–0.926 in the mixed-vintage family at 1:10. At 1:5, the corresponding ranges are 0.827–0.908 and 0.891–0.936. PMGN and CWAN method coverage checks at 1:10 show that the main deployment directions are not driven only by a single selected method, although C6 remains a borderline method-sensitive case.

The results also show why the raw Macro-F1 alone is not sufficient. GapClosure reveals how much of the source-only-to-oracle interval is recovered, while the matched-budget target-only comparison reveals whether adaptation is the best use of the available labelled target subset. An improvement over source-only is therefore necessary but not sufficient for deployment justification. In some contexts, native DA exceeds matched-budget target-only learning; in others, target-side supervised learning dominates; and, in crossover cases, DA is most useful under severe label scarcity but is overtaken as the target label budget grows.

The representation contract analysis further shows that feature harmonisation is part of the empirical claim. Intersection and Union change the realised difficulty of a context. Union penalises GGA in SS-HDA, benefits JSTN in the contemporary-source MS-HDA tuple, and penalises JSTN in the mixed-vintage tuple. These results show that representation contracts should be reported as first-class benchmark components rather than treated as hidden preprocessing details.

The ToN-IoT confirmatory experiment further supports a target-conditioned interpretation. It shows that the headroom recovery framework remains meaningful under a second IoT/IIoT target, while also confirming that the absolute GapClosure magnitudes and same-budget deployment directions remain target-dependent. The result is therefore not a universal target-independent ranking but an auditable way to interpret adaptation evidence within declared target contexts.

The overall conclusion is therefore precise: the evaluated primary native closed-set HDA methods can recover a large share of target supervision headroom in both single-source and multi-source IoT IDS transfer, but their deployment value is target-conditioned and must be judged within the resolved context and against matched-budget target-only learning. The paper contributes not only a set of closed-set results but also an auditable interpretation scale: the raw Macro-F1 establishes the achieved performance, GapClosure measures the recovered headroom, paired statistical tests quantify seed-level uncertainty in DA-versus-target-only differences, matched-budget target-only learning tests the deployment alternative, and residual headroom identifies the remaining value of target-native supervision.

Future work could extend the benchmark to additional source–target families, heterogeneous target settings, multi-target or time-evolving target adaptation, richer attack taxonomies, more native HDA methods, and the unsupervised and open-set settings excluded here. These extensions would test whether the observed headroom recovery patterns, representation sensitivities, same-budget deployment decisions, and method coverage findings persist under broader IDS transfer conditions and across changing target environments.

## Figures and Tables

**Table 1:** Positioning of the present paper relative to related work.

Research Strand	Representative Works	Main Contribution of the Strand	How the Present Paper Extends It
Closed-set HDA methods	GGA [16]; JSTN [17]; PMGN [18]; CWAN [19]	Develop native methods for heterogeneous transfer under limited target supervision, prototype-based cross-domain matching, or multi-source source-side supervision.	Evaluates primary selected native SS-HDA and MS-HDA exemplars under fixed contexts, labelled target budgets, representation contracts, and anchor references; uses PMGN and CWAN as 1:10 method coverage checks to reduce dependence on a single selected method.
Unsupervised and open-set DA for IDS	GWR-GCN [23]; OSDN [24]; OSDA work [25]	Addresses settings where target labels are unavailable or where unknown target classes may appear.	Recognises these as important but distinct settings with different supervision contracts and output semantics; avoids mixing them with closed-set claims.
Heterogeneous IDS datasets and benchmark resources	CICIDS2017 [26]; UNSW-NB15 [27]; NSL-KDD [7]; ToN-IoT [21]; Edge-IIoTset [22]; TriHID [15]	Provide realistic source and target domains for studying heterogeneous IDS transfer.	Uses multiple source configurations with Edge-IIoTset as the main fixed target and ToN-IoT as a compact second-target confirmatory check, while making label contracts, feature contracts, split identities, and target-conditioned interpretation explicit.
Cross-dataset IDS evaluation discipline	Apruzzese et al. [6]; Chou et al. [14]; Jin et al. [5]	Shows that dataset choice, protocol design, and evaluation context can materially change conclusions.	Operationalises this concern through fixed resolved contexts, leakage-safe preprocessing, repeated seeds, paired statistical testing, admissibility checks, and anchor-based interpretation.
Anchor-based DA benchmarking	AdaTime [28]; multi-source DA benchmarks [29]	Uses source-only and target-aware references to contextualise adaptation gains.	Extends anchor logic into a closed-set IoT IDS headroom analysis using GapClosure, matched-budget target-only comparison, and deployment direction interpretation.

The table is interpretive rather than exhaustive. It identifies the strands most directly connected to the closed-set benchmark developed in this paper.

**Table 2:** Closed-set label contract resolution used in the benchmark.

Dataset Family	Benchmark Classes Retained	Merged Raw Families	Excluded Families or Notes
CICIDS2017	normal, scan, DoS, DDoS, exploit/credential abuse	DoS subtypes are merged into DoS; credential and application abuse behaviours are merged into exploit/credential abuse	Bot, Infiltration and Heartbleed are excluded from the closed-set contract because they do not provide stable cross-dataset alignment in the reported tuples
UNSW-NB15	normal, scan, DoS, DDoS, exploit/credential abuse	Generic is mapped into DDoS, where it functions as high-volume disruptive traffic; exploits are mapped into exploit/credential abuse	Families with weak semantic alignment to the target contract are excluded rather than retained as unstable benchmark classes
NSL-KDD	normal, scan, DoS, DDoS, exploit/credential abuse	Probe is mapped to scan; DoS variants are mapped to DoS/DDoS according to benchmark-side consolidation; U2R/R2L-style behaviours are mapped into exploit/credential abuse where retained	Used only as a legacy auxiliary source in MS-HDA; not interpreted as a modern IoT source by itself
Edge-IIoTset	normal, scan, DoS, DDoS, exploit/credential abuse	Target attack families are consolidated into the five benchmark classes where alignment is credible	Target-side families outside the closed-set contract are excluded from this paper rather than treated as unknown classes
ToN-IoT confirmatory target	normal, scan, DoS, DDoS, exploit/credential abuse	injection, password, and xss are merged into exploit/credential abuse	backdoor, ransomware, and mitm are excluded from the closed-set confirmatory contract; the full ToN-IoT mapping is reported in Appendix E

The table reports the benchmark-level resolution logic. Exact raw-label strings for the Edge-IIoTset benchmark and the ToN-IoT confirmatory target are listed in the Appendix and repository manifest. The reported Macro-F1 and GapClosure values are therefore conditioned on the declared five-class contract and should not be compared directly with studies using a richer or different attack taxonomy.

**Table 3:** Realised feature-space cardinalities under the closed-set Intersection and Union representation contracts.

Domain Tuple	Protocol	Shared Raw Candidates	Retained Final Features	Numeric	Categorical	Union-Only Materialised	Excluded
CICIDS2017 → Edge-IIoTset	Intersection	26	22	20	2	0	11
CICIDS2017 → Edge-IIoTset	Union	26	34	29	5	12	11
UNSW-NB15 → Edge-IIoTset	Intersection	21	17	15	2	0	16
UNSW-NB15 → Edge-IIoTset	Union	21	33	27	6	16	16
CICIDS2017 + UNSW-NB15 → Edge-IIoTset	Intersection	18	14	12	2	0	19
CICIDS2017 + UNSW-NB15 → Edge-IIoTset	Union	18	36	29	7	22	19
CICIDS2017 + NSL-KDD → Edge-IIoTset	Intersection	19	16	13	3	0	20
CICIDS2017 + NSL-KDD → Edge-IIoTset	Union	19	31	25	6	15	20

The arrow (→) denotes the source-to-target transfer direction. “Shared raw candidates” records features examined as potentially admissible before exclusions. “Retained final features” records the actual execution interface after protocol-specific realisation. Union-only materialised features are absent from at least one domain and are inserted using the declared materialisation rule. The “Excluded” column counts exclusion entries across the datasets in a tuple, including identifier-like, timestamp-derived, label-proximal, protocol-specific, endpoint, and record management fields. It is not simply the arithmetic difference between shared raw candidates and retained final features. Union-only materialised fields are retained executable features and are therefore not counted as exclusions.

**Table 4:** Closed-set benchmark contexts. SO = source-only; DA = evaluated domain adaptation method; TO = target-only supervised reference.

Ctx	Domain Tuple	Setting	Protocol	Ratio	Target Test	Main Mismatch Source	Permitted References
C1	CICIDS2017 → Edge-IIoTset	SS-HDA	Intersection	R	200,000	Environment shift and feature interface mismatch	SO, CORAL, DA, TO
C2	CICIDS2017 → Edge-IIoTset	SS-HDA	Union	R	200,000	Environment shift and materialised schema mismatch	SO, CORAL, DA, TO
C3	UNSW-NB15 → Edge-IIoTset	SS-HDA	Intersection	R	200,000	Collection process shift and feature interface mismatch	SO, CORAL, DA, TO
C4	UNSW-NB15 → Edge-IIoTset	SS-HDA	Union	R	200,000	Collection process shift and materialised schema mismatch	SO, CORAL, DA, TO
C5	CICIDS2017 + UNSW-NB15 → Edge-IIoTset	MS-HDA	Intersection	R	200,000	Contemporary source diversity and target mismatch	SO, CORAL, DA, TO
C6	CICIDS2017 + UNSW-NB15 → Edge-IIoTset	MS-HDA	Union	R	200,000	Contemporary source diversity under materialised schema mismatch	SO, CORAL, DA, TO
C7	CICIDS2017 + NSL-KDD → Edge-IIoTset	MS-HDA	Intersection	R	200,000	Mixed-vintage source divergence and target mismatch	SO, CORAL, DA, TO
C8	CICIDS2017 + NSL-KDD → Edge-IIoTset	MS-HDA	Union	R	200,000	Mixed-vintage source divergence under materialised schema mismatch	SO, CORAL, DA, TO

The arrow (→) denotes the source-to-target transfer direction. The target test identity is fixed within each context family. The labelled target ratios define the size of the labelled subset drawn from the target training pool, not the target test set. The same context is never pooled across ratios or representation protocols.

**Table 5:** Edge-IIoTset target-side labelled budget cardinalities used in C1–C8.

Ratio	Target Labelled	Target Unlabelled	Target Test
1:50	2000	100,000	200,000
1:10	10,000	100,000	200,000
1:5	20,000	100,000	200,000

The same Edge-IIoTset target budget convention is used across C1–C8. Source training cardinalities differ by tuple and are reported in the split manifest and reproducibility appendix. ToN-IoT confirmatory cardinalities are reported separately in Appendix E.

**Table 6:** ToN-IoT second-target confirmatory contexts.

Ctx.	Source Domain (s)	Target	Regime	Protocol	Ratio	Method
T1	C17	ToN-IoT	SS-HDA	Intersection	1:10	GGA
T2	C17	ToN-IoT	SS-HDA	Union	1:10	GGA
T3	C17 + U15	ToN-IoT	MS-HDA	Intersection	1:10	JSTN
T4	C17 + U15	ToN-IoT	MS-HDA	Union	1:10	JSTN

C17 denotes CICIDS2017 and U15 denotes UNSW-NB15. All four rows are second-target confirmatory checks rather than additional full-benchmark contexts. They use the same closed-set label contract principle, leakage-safe preprocessing rule, twenty-seed policy, GapClosure definition, and matched-budget target-only comparison as the main Edge-IIoTset benchmark, but they are analysed separately and are not pooled with C1–C8.

**Table 7:** Closed-set methods and declared benchmark roles.

Method	Benchmark Role	Source Setting	Target Supervision Contract	Evaluated Contexts	Reporting Role
GGA	Primary SS-HDA exemplar	One labelled source domain	$D_{T,r}^{L}+D_{T,\mathrm{train}}^{U}$	C1–C4, all ratios; T1–T2 at 1:10	Primary result
PMGN	Secondary SS-HDA coverage method	One labelled source domain	$D_{T,1:10}^{L}+D_{T,\mathrm{train}}^{U}$	C1–C4 at 1:10	Coverage check
JSTN	Primary MS-HDA exemplar	Multiple labelled source domains	$D_{T,r}^{L}+D_{T,\mathrm{train}}^{U}$	C5–C8, all ratios; T3–T4 at 1:10	Primary result
CWAN	Secondary MS-HDA coverage method	Multiple labelled source domains	$D_{T,1:10}^{L}+D_{T,\mathrm{train}}^{U}$	C5–C8 at 1:10	Coverage check

$D_{T,r}^{L}$ denotes the ratio-specific labelled target subset for ratio r∈{1:50, 1:10, 1:5}, and $D_{T,\mathrm{train}}^{U}$ denotes the unlabelled target-training pool permitted by the declared closed-set context. For PMGN and CWAN, r=1:10 because they are used only as method coverage checks at the moderate labelled target budget. GGA and JSTN provide the primary selected native exemplars for the SS-HDA and MS-HDA analyses, respectively. The ToN-IoT contexts T1–T4 are second-target confirmatory checks and are not pooled with the main Edge-IIoTset contexts C1–C8. All rows use the declared closed-set label contract, leakage-safe preprocessing, fixed target test identity, and the corresponding Intersection or Union representation contract. The fixed target test split is never used for training, adaptation, model selection, threshold selection, or post hoc calibration.

**Table 8:** Anchor ladder used to interpret closed-set HDA performance.

Anchor	Role in This Paper	Target Label Use	Ratio Dependence
Source-only	Naive cross-domain transfer floor without adaptation	No target labels	Fixed within context family
CORAL	Lightweight second-order alignment reference under the same split and representation contract	No target labels; uses unlabelled target-training features	Fixed within context family
Target-only, matched-budget	Direct supervised target reference using the same labelled target budget available to the DA method	Uses ratio-specific labelled target subset	Ratio-specific
Target-only, oracle	Upper target-native reference using the full legal target training pool	Uses full legal target training labels	Fixed within context family

The anchor ladder is source-only → CORAL → native DA method → target-only references. The matched-budget target-only anchor answers whether DA is competitive with direct supervised learning under the same target label budget. The oracle target-only anchor defines the full target supervision reference used in GapClosure.

**Table 9:** Run policy and implementation disclosure for the closed-set benchmark.

Item	Value
Repeated-run policy	Twenty runs per fixed method–context–ratio configuration, with the same seed determining model initialisation and labelled target subsampling.
Seed list	{11, 17, 23, 31, 47, 53, 59, 67, 71, 79, 83, 89, 97, 101, 103, 107, 109, 113, 127, 131}.
Aggregation rule	Mean ± standard deviation across twenty seeds within fixed context, ratio, representation contract, and declared method role. Paired DA-versus-target-only statistical tests are reported separately.
Primary SS-HDA exemplar	GGA in C1–C4 across 1:50, 1:10, and 1:5
Secondary SS-HDA coverage check	PMGN in C1–C4 at 1:10
Primary MS-HDA exemplar	JSTN in C5–C8 across 1:50, 1:10, and 1:5
Secondary MS-HDA coverage check	CWAN in C5–C8 at 1:10
Neural optimiser	Adam
Learning rate	$1\times 10^{-3}$
Weight decay	$1\times 10^{-4}$
Mini-batch size	256
Maximum epochs	100
Early stopping patience	15 epochs
Closed-set model selection metric	Validation Macro-F1 on context-permitted validation data
Target test use	Never used for training, preprocessing fit, hyperparameter selection, threshold choice, model selection, or post hoc calibration
Anchor learner	LightGBM multiclass with fixed benchmark settings for source-only, CORAL, matched-budget target-only, and oracle target-only rows
Admissibility requirement	Split identity, label contract, representation protocol, leakage boundary, method role flag, predictions, metrics, and provenance must be complete before aggregation

**Table 10:** Anchor overview across closed-set contexts.

Ctx	Domain Tuple	Setting	Protocol	Source-Only Macro-F1	CORAL Macro-F1	Matched-Budget Target-Only Range	Oracle Target-Only
C1	CIC17 → Edge	SS-HDA	Intersection	0.558	0.669	0.829–0.901	0.940
C2	CIC17 → Edge	SS-HDA	Union	0.541	0.642	0.806–0.862	0.931
C3	UNSW → Edge	SS-HDA	Intersection	0.487	0.595	0.801–0.874	0.940
C4	UNSW → Edge	SS-HDA	Union	0.469	0.570	0.778–0.858	0.931
C5	CIC17+UNSW → Edge	MS-HDA	Intersection	0.561	0.677	0.857–0.931	0.950
C6	CIC17+UNSW → Edge	MS-HDA	Union	0.551	0.677	0.854–0.915	0.940
C7	CIC17+NSL → Edge	MS-HDA	Intersection	0.518	0.635	0.861–0.916	0.949
C8	CIC17+NSL → Edge	MS-HDA	Union	0.500	0.612	0.859–0.923	0.941

CIC17 = CICIDS2017; UNSW = UNSW-NB15; NSL = NSL-KDD; Edge = Edge-IIoTset. All point values represent means across the twenty fixed seeds. The matched-budget target-only column reports the observed range of ratio-specific means across 1:50, 1:10, and 1:5. Source-only, CORAL, and oracle target-only are ratio-invariant within a context family under the fixed context definition. The ratio-specific matched-budget values are used explicitly in Section 5.

**Table 11:** Native SS-HDA performance of GGA across single-source contexts.

Ctx	Tuple	Protocol	Ratio	Transfer Anchors		Primary Native DA	Target Ref.
				Src.	CORAL	GGA	Oracle
C1	CICIDS2017 → Edge-IIoTset	Intersection	1:50	0.558	0.669	0.855±0.006	0.940
C1	CICIDS2017 → Edge-IIoTset	Intersection	1:10	0.558	0.669	0.877±0.008	0.940
C1	CICIDS2017 → Edge-IIoTset	Intersection	1:5	0.558	0.669	0.885±0.004	0.940
C2	CICIDS2017 → Edge-IIoTset	Union	1:50	0.541	0.642	0.791±0.006	0.931
C2	CICIDS2017 → Edge-IIoTset	Union	1:10	0.541	0.642	0.816±0.005	0.931
C2	CICIDS2017 → Edge-IIoTset	Union	1:5	0.541	0.642	0.824±0.008	0.931
C3	UNSW-NB15 → Edge-IIoTset	Intersection	1:50	0.487	0.595	0.782±0.008	0.940
C3	UNSW-NB15 → Edge-IIoTset	Intersection	1:10	0.487	0.595	0.814±0.008	0.940
C3	UNSW-NB15 → Edge-IIoTset	Intersection	1:5	0.487	0.595	0.824±0.007	0.940
C4	UNSW-NB15 → Edge-IIoTset	Union	1:50	0.469	0.570	0.731±0.007	0.931
C4	UNSW-NB15 → Edge-IIoTset	Union	1:10	0.469	0.570	0.762±0.009	0.931
C4	UNSW-NB15 → Edge-IIoTset	Union	1:5	0.469	0.570	0.776±0.008	0.931

All numeric values are Macro-F1. *Src.* denotes the source-only transfer baseline, CORAL denotes the lightweight alignment anchor, and Oracle denotes the oracle target-only reference. GGA is reported as mean ± standard deviation over the twenty fixed seeds and is interpreted only as the primary selected SS-HDA exemplar. Matched-budget target-only values are omitted from this compact table and used explicitly in Section 5.

**Table 12:** Native MS-HDA performance of JSTN across multi-source contexts.

Ctx	Tuple	Protocol	Ratio	Transfer Anchors		Primary Native DA	Target Ref.
				Src.	CORAL	JSTN	Oracle
C5	CICIDS2017 + UNSW-NB15 → Edge-IIoTset	Intersection	1:50	0.561	0.677	0.840±0.009	0.950
C5	CICIDS2017 + UNSW-NB15 → Edge-IIoTset	Intersection	1:10	0.561	0.677	0.863±0.008	0.950
C5	CICIDS2017 + UNSW-NB15 → Edge-IIoTset	Intersection	1:5	0.561	0.677	0.883±0.005	0.950
C6	CICIDS2017 + UNSW-NB15 → Edge-IIoTset	Union	1:50	0.551	0.677	0.877±0.009	0.940
C6	CICIDS2017 + UNSW-NB15 → Edge-IIoTset	Union	1:10	0.551	0.677	0.900±0.006	0.940
C6	CICIDS2017 + UNSW-NB15 → Edge-IIoTset	Union	1:5	0.551	0.677	0.904±0.007	0.940
C7	CICIDS2017 + NSL-KDD → Edge-IIoTset	Intersection	1:50	0.518	0.635	0.888±0.009	0.949
C7	CICIDS2017 + NSL-KDD → Edge-IIoTset	Intersection	1:10	0.518	0.635	0.917±0.010	0.949
C7	CICIDS2017 + NSL-KDD → Edge-IIoTset	Intersection	1:5	0.518	0.635	0.922±0.009	0.949
C8	CICIDS2017 + NSL-KDD → Edge-IIoTset	Union	1:50	0.500	0.612	0.853±0.007	0.941
C8	CICIDS2017 + NSL-KDD → Edge-IIoTset	Union	1:10	0.500	0.612	0.885±0.004	0.941
C8	CICIDS2017 + NSL-KDD → Edge-IIoTset	Union	1:5	0.500	0.612	0.893±0.008	0.941

All numeric values are Macro-F1. *Src.* denotes the source-only transfer baseline, CORAL denotes the lightweight alignment anchor, and Oracle denotes the oracle target-only reference. JSTN is reported as mean ± standard deviation over the twenty fixed seeds and is interpreted only as the primary selected MS-HDA exemplar. Matched-budget target-only values are omitted from this compact table and used explicitly in Section 5.

**Table 13:** Representation contract sensitivity of native closed-set DA.

Tuple Family	Regime	Method	Ratio	Macro-F1 by Representation Contract		
				I	U	$\Delta_{U-I}$
CICIDS2017 → Edge-IIoTset	SS-HDA	GGA	1:50	0.855	0.791	−0.064
CICIDS2017 → Edge-IIoTset	SS-HDA	GGA	1:10	0.877	0.816	−0.060
CICIDS2017 → Edge-IIoTset	SS-HDA	GGA	1:5	0.885	0.824	−0.061
UNSW-NB15 → Edge-IIoTset	SS-HDA	GGA	1:50	0.782	0.731	−0.051
UNSW-NB15 → Edge-IIoTset	SS-HDA	GGA	1:10	0.814	0.762	−0.052
UNSW-NB15 → Edge-IIoTset	SS-HDA	GGA	1:5	0.824	0.776	−0.049
CICIDS2017 + UNSW-NB15 → Edge-IIoTset	MS-HDA	JSTN	1:50	0.840	0.877	+0.037
CICIDS2017 + UNSW-NB15 → Edge-IIoTset	MS-HDA	JSTN	1:10	0.863	0.900	+0.037
CICIDS2017 + UNSW-NB15 → Edge-IIoTset	MS-HDA	JSTN	1:5	0.883	0.904	+0.022
CICIDS2017 + NSL-KDD → Edge-IIoTset	MS-HDA	JSTN	1:50	0.888	0.853	−0.035
CICIDS2017 + NSL-KDD → Edge-IIoTset	MS-HDA	JSTN	1:10	0.917	0.885	−0.033
CICIDS2017 + NSL-KDD → Edge-IIoTset	MS-HDA	JSTN	1:5	0.922	0.893	−0.028

I denotes the Intersection representation contract and U denotes the Union representation contract. All values are mean Macro-F1 scores. $\Delta_{U-I}$ is computed as Union minus Intersection for the same tuple family, regime, native method, and labelled target ratio. Negative values indicate that Union reduces the Macro-F1 relative to Intersection; positive values indicate that Union improves the Macro-F1 relative to Intersection.

**Table 14:** Label budget and seed stability profile for primary native closed-set DA.

Summary Group	Method	Ratio	Mean Macro-F1 Across Contexts	Context-Level Range	Mean Seed-Level std
SS-HDA, C1–C4	GGA	1:50	0.790	0.731–0.855	0.007
SS-HDA, C1–C4	GGA	1:10	0.817	0.762–0.877	0.008
SS-HDA, C1–C4	GGA	1:5	0.827	0.776–0.885	0.007
MS-HDA contemporary-source, C5–C6	JSTN	1:50	0.858	0.840–0.877	0.009
MS-HDA contemporary-source, C5–C6	JSTN	1:10	0.881	0.863–0.900	0.007
MS-HDA contemporary-source, C5–C6	JSTN	1:5	0.894	0.883–0.904	0.006
MS-HDA mixed-vintage, C7–C8	JSTN	1:50	0.870	0.853–0.888	0.008
MS-HDA mixed-vintage, C7–C8	JSTN	1:10	0.901	0.885–0.917	0.007
MS-HDA mixed-vintage, C7–C8	JSTN	1:5	0.907	0.893–0.922	0.008

The SS-HDA rows average GGA across C1–C4. The MS-HDA rows are stratified by source-pair family: C5–C6 correspond to the contemporary source pair, while C7–C8 correspond to the mixed-vintage pair. The context-level range reports the lowest and highest context mean within each declared group and ratio. The mean seed-level standard deviation averages the twenty-seed standard deviations for the same rows.

**Table 15:** Per-context GapClosure for primary native closed-set DA.

Ctx	Tuple	Regime	Primary Native Method	1:50	1:10	1:5
C1	CICIDS2017 → Edge-IIoTset	SS-HDA	GGA	0.778	0.835	0.857
C2	CICIDS2017 → Edge-IIoTset	SS-HDA	GGA	0.642	0.707	0.727
C3	UNSW-NB15 → Edge-IIoTset	SS-HDA	GGA	0.651	0.722	0.745
C4	UNSW-NB15 → Edge-IIoTset	SS-HDA	GGA	0.566	0.633	0.663
C5	CICIDS2017 + UNSW-NB15 → Edge-IIoTset	MS-HDA	JSTN	0.718	0.776	0.827
C6	CICIDS2017 + UNSW-NB15 → Edge-IIoTset	MS-HDA	JSTN	0.837	0.897	0.908
C7	CICIDS2017 + NSL-KDD → Edge-IIoTset	MS-HDA	JSTN	0.858	0.926	0.936
C8	CICIDS2017 + NSL-KDD → Edge-IIoTset	MS-HDA	JSTN	0.800	0.872	0.891

GapClosure is computed as $\left(\Phi \left(m,c,r\right)-\Phi_{\mathrm{SO}}\left(c\right)\right)/\left(\Phi_{\mathrm{TO}\text{-}\mathrm{oracle}}\left(c\right)-\Phi_{\mathrm{SO}}\left(c\right)\right)$, using Macro-F1 means across the twenty fixed seeds. Values are rounded to three decimals.

**Table 16:** Regime family GapClosure summaries for primary native closed-set DA.

Summary Group	Contexts	Primary Native Method	Ratio	Descriptive Mean GapClosure	Between-Context Spread
SS-HDA, CICIDS2017 source	C1–C2	GGA	1:50	0.710	0.096
SS-HDA, CICIDS2017 source	C1–C2	GGA	1:10	0.771	0.091
SS-HDA, CICIDS2017 source	C1–C2	GGA	1:5	0.792	0.092
SS-HDA, UNSW-NB15 source	C3–C4	GGA	1:50	0.609	0.060
SS-HDA, UNSW-NB15 source	C3–C4	GGA	1:10	0.678	0.063
SS-HDA, UNSW-NB15 source	C3–C4	GGA	1:5	0.704	0.058
MS-HDA, contemporary-source	C5–C6	JSTN	1:50	0.778	0.084
MS-HDA, contemporary-source	C5–C6	JSTN	1:10	0.837	0.086
MS-HDA, contemporary-source	C5–C6	JSTN	1:5	0.868	0.057
MS-HDA, mixed-vintage	C7–C8	JSTN	1:50	0.829	0.041
MS-HDA, mixed-vintage	C7–C8	JSTN	1:10	0.899	0.038
MS-HDA, mixed-vintage	C7–C8	JSTN	1:5	0.914	0.032

The descriptive mean and between-context spread are computed from context-level GapClosure values within each declared source-tuple family. Each two-context family shares the same source–target tuple and differs only in representation contract: Intersection versus Union. The summary therefore describes representation sensitivity within a coherent tuple rather than cross-tuple pooling. These values are descriptive only and are not used as pooled performance estimates or inferential statistics.

**Table 17:** Sign-based same-budget comparison between native DA and matched-budget target-only learning.

Summary Group	Contexts	Ratio	DA > Target Only	Approx. Tie	DA < Target Only
SS-HDA, CICIDS2017 source	C1–C2	1:50	1/2	0/2	1/2
SS-HDA, CICIDS2017 source	C1–C2	1:10	0/2	1/2	1/2
SS-HDA, CICIDS2017 source	C1–C2	1:5	0/2	0/2	2/2
SS-HDA, UNSW-NB15 source	C3–C4	1:50	0/2	0/2	2/2
SS-HDA, UNSW-NB15 source	C3–C4	1:10	0/2	0/2	2/2
SS-HDA, UNSW-NB15 source	C3–C4	1:5	0/2	0/2	2/2
MS-HDA, contemporary-source	C5–C6	1:50	1/2	0/2	1/2
MS-HDA, contemporary-source	C5–C6	1:10	0/2	1/2	1/2
MS-HDA, contemporary-source	C5–C6	1:5	0/2	0/2	2/2
MS-HDA, mixed-vintage	C7–C8	1:50	1/2	1/2	0/2
MS-HDA, mixed-vintage	C7–C8	1:10	1/2	0/2	1/2
MS-HDA, mixed-vintage	C7–C8	1:5	0/2	1/2	1/2

This table summarises the signs of $\Delta_{r}={\mathrm{GapClosure}}_{DA}\left(c,r\right)-{\mathrm{GapClosure}}_{matched\text{-}TO}\left(c,r\right)$ from Table 18. A positive sign indicates that native DA recovers more source-only-to-oracle headroom than matched-budget target-only learning at the same labelled target ratio. An approximate tie is defined as $|\Delta_{r}|\leq 0.02$. The threshold is used as an operational tolerance for small GapClosure differences relative to three-decimal reporting precision and twenty-seed paired variability. Each summary group contains paired contexts that share the same source–target tuple and differ only by representation contract: Intersection versus Union. The table reports sign incidence only and does not average GapClosure values across heterogeneous source–target tuples.

**Table 18:** Same-ratio GapClosure difference between native DA and matched-budget target-only learning.

Ctx	Regime	Tuple	$\Delta_{1:50}$	$\Delta_{1:10}$	$\Delta_{1:5}$
C1	SS-HDA	CICIDS2017 → Edge-IIoTset	+0.067	−0.002	−0.041
C2	SS-HDA	CICIDS2017 → Edge-IIoTset	−0.037	−0.045	−0.097
C3	SS-HDA	UNSW-NB15 → Edge-IIoTset	−0.042	−0.070	−0.110
C4	SS-HDA	UNSW-NB15 → Edge-IIoTset	−0.102	−0.152	−0.178
C5	MS-HDA	CICIDS2017 + UNSW-NB15 → Edge-IIoTset	−0.044	−0.129	−0.125
C6	MS-HDA	CICIDS2017 + UNSW-NB15 → Edge-IIoTset	+0.058	−0.005	−0.028
C7	MS-HDA	CICIDS2017 + NSL-KDD → Edge-IIoTset	+0.062	+0.058	+0.013
C8	MS-HDA	CICIDS2017 + NSL-KDD → Edge-IIoTset	−0.013	−0.069	−0.067

$\Delta_{r}={\mathrm{GapClosure}}_{\mathrm{DA}}\left(c,r\right)-{\mathrm{GapClosure}}_{\mathrm{matched}\text{-}\mathrm{TO}}\left(c,r\right)$. Positive values indicate that native DA recovers more source-to-oracle headroom than matched-budget target-only learning at the same labelled target ratio. Negative values indicate that direct target-side supervised learning recovers more headroom under the same label budget. Values are dimensionless GapClosure differences, not Macro-F1 differences.

**Table 19:** Selected paired statistical comparisons between DA and matched-budget target-only learning.

Analysis Block	Ctx.	Method	Protocol	Ratio	Mean Paired ΔF1 [95% CI]	Adj. $p_{t}$	Adj. $p_{W}$	$d_{z}$	$r_{\mathrm{rb}}$
*Edge-IIoTset primary native DA examples*									
SS-HDA primary	C1	GGA	Intersection	1:10	−0.001 [−0.003,0.002]	0.601	0.240	−0.119	−0.368
SS-HDA primary	C4	GGA	Union	1:10	−0.070 [−0.073,−0.068]	<0.001	<0.001	−12.066	−1.000
MS-HDA primary	C7	JSTN	Intersection	1:10	+0.025 [0.023,0.028]	<0.001	<0.001	4.388	1.000
MS-HDA primary	C8	JSTN	Union	1:10	−0.031 [−0.033,−0.028]	<0.001	<0.001	−5.663	−1.000
*Edge-IIoTset 1:10 method coverage examples*									
SS-HDA coverage	C1	PMGN	Intersection	1:10	−0.006 [−0.008,−0.004]	<0.001	<0.001	−1.122	−0.980
MS-HDA coverage	C7	CWAN	Intersection	1:10	+0.018 [0.016,0.020]	<0.001	<0.001	4.212	1.000
*ToN-IoT second-target confirmatory examples*									
SS-HDA confirmatory	T2	GGA	Union	1:10	−0.012 [−0.015,−0.009]	<0.001	<0.001	−1.648	−0.971
MS-HDA confirmatory	T3	JSTN	Intersection	1:10	+0.019 [0.016,0.022]	<0.001	<0.001	2.805	1.000

ΔF1 is DA Macro-F1 minus matched-budget target-only Macro-F1 under the same seed, context, ratio, representation contract, and labelled target subset. The mean paired ΔF1 column reports the bootstrap 95% confidence interval in brackets. $p_{t}$ is the paired *t*-test *p*-value and $p_{W}$ is the exact Wilcoxon signed-rank *p*-value; both are Holm–Bonferroni-adjusted within their declared inference family. $d_{z}$ is Cohen’s paired-samples effect size and $r_{rb}$ is the matched-pairs rank-biserial correlation. The table reports selected main text examples only; the full 36-cell paired statistical table is reported in Appendix F.

**Table 20:** The 1:10 method coverage checks for PMGN and CWAN.

Ctx	Regime	Protocol	Primary Method	Primary GapClosure	Coverage Method	Coverage GapClosure
C1	SS-HDA	Intersection	GGA	0.835	PMGN	0.821
C2	SS-HDA	Union	GGA	0.707	PMGN	0.686
C3	SS-HDA	Intersection	GGA	0.722	PMGN	0.701
C4	SS-HDA	Union	GGA	0.633	PMGN	0.606
C5	MS-HDA	Intersection	JSTN	0.776	CWAN	0.812
C6	MS-HDA	Union	JSTN	0.897	CWAN	0.927
C7	MS-HDA	Intersection	JSTN	0.926	CWAN	0.910
C8	MS-HDA	Union	JSTN	0.872	CWAN	0.854

PMGN and CWAN are included as 1:10 method coverage checks. Their results are interpreted within the same resolved contexts as the primary methods but are not averaged with those of GGA or JSTN.

**Table 21:** The 1:10 deployment direction agreement between primary and coverage methods.

Ctx	Regime	Primary $\Delta_{1:10}$	Coverage $\Delta_{1:10}$	Direction Agreement	Interpretation
C1	SS-HDA	−0.002	−0.016	Agree	Both methods obtain approximate ties with matched-budget target-only.
C2	SS-HDA	−0.045	−0.067	Agree	Both methods fall below target-only under the Union stress condition.
C3	SS-HDA	−0.070	−0.090	Agree	Both methods fall below target-only; PMGN is slightly weaker.
C4	SS-HDA	−0.152	−0.180	Agree	Both methods show target-only dominance in the hardest SS-HDA setting.
C5	MS-HDA	−0.129	−0.093	Agree	Both methods remain below target-only; CWAN is closer.
C6	MS-HDA	−0.005	+0.025	Diverge	Borderline: JSTN is tied, while CWAN is slightly above target-only.
C7	MS-HDA	+0.058	+0.042	Agree	Both methods support a DA-favourable deployment direction.
C8	MS-HDA	−0.069	−0.087	Agree	Both methods remain below the strong target-only reference under Union.

$\Delta_{1:10}$ is computed as the method GapClosure minus the matched-budget target-only GapClosure in the same context. Direction agreement means that the primary and coverage methods fall on the same side of the matched-budget target-only reference, using the $|\Delta_{1:10}|\leq 0.02$ approximate tie tolerance.

**Table 22:** ToN-IoT second-target confirmatory results at the 1:10 labelled target ratio.

Ctx	Target	Regime	Protocol	Method	DA Macro-F1	DA GapClosure	$\Delta_{1:10}$	Direction
T1	ToN-IoT	SS-HDA	Intersection	GGA	0.782±0.031	0.650	+0.036	DA > TO
T2	ToN-IoT	SS-HDA	Union	GGA	0.778±0.031	0.667	−0.034	DA < TO
T3	ToN-IoT	MS-HDA	Intersection	JSTN	0.804±0.032	0.680	+0.058	DA > TO
T4	ToN-IoT	MS-HDA	Union	JSTN	0.792±0.028	0.675	−0.014	Tie

$\Delta_{1:10}$ is computed as DA GapClosure minus matched-budget target-only GapClosure under the same ToN-IoT context. Positive values indicate that DA recovers more source-only-to-oracle headroom than direct target-only learning under the same labelled target budget. A tie uses the same |Δ|≤0.02 operational tolerance as the Edge-IIoTset analysis. The corresponding paired statistical tests for the ToN-IoT confirmatory contexts are reported with the full paired-test outputs in Appendix F.

**Table 23:** Residual source-only-to-oracle headroom after primary native closed-set DA.

Summary Group	Contexts	Primary Native Method	Ratio	Mean Residual	Between-Context Spread
SS-HDA	C1–C4	GGA	1:50	0.341	0.088
SS-HDA	C1–C4	GGA	1:10	0.276	0.083
SS-HDA	C1–C4	GGA	1:5	0.252	0.081
MS-HDA, contemporary-source	C5–C6	JSTN	1:50	0.222	0.084
MS-HDA, contemporary-source	C5–C6	JSTN	1:10	0.163	0.086
MS-HDA, contemporary-source	C5–C6	JSTN	1:5	0.132	0.057
MS-HDA, mixed-vintage	C7–C8	JSTN	1:50	0.171	0.041
MS-HDA, mixed-vintage	C7–C8	JSTN	1:10	0.101	0.038
MS-HDA, mixed-vintage	C7–C8	JSTN	1:5	0.086	0.032

Residual headroom is 1−GapClosure. Lower values indicate that the primary native DA method has recovered more of the source-only-to-oracle interval. The descriptive mean and between-context spread are computed only within the declared regime family group and are not used as pooled cross-context performance estimates. For the MS-HDA summaries, C5–C6 and C7–C8 are kept separate because they correspond to different source-pair families.

## Data Availability

Data are available on request from the corresponding author.

## Abbreviations

The following abbreviations are used in this manuscript:

DA	Domain adaptation
HDA	Heterogeneous domain adaptation
SS-HDA	Single-source semi-supervised heterogeneous domain adaptation
MS-HDA	Multi-source semi-supervised heterogeneous domain adaptation
IoT	Internet of Things
IIoT	Industrial Internet of Things
IDS	Intrusion detection system
NIDS	Network intrusion detection system
GGA	Geometric Graph Alignment
JSTN	Joint Semantic Transfer Network
PMGN	Prototype-Matching Graph Network
CWAN	Conditional Weighting Adversarial Network
CORAL	Correlation alignment
Macro-F1	Macro-averaged F1-score
ROC-AUC	Area under the receiver operating characteristic curve
PR-AUC	Area under the precision–recall curve
OSDA	Open-set domain adaptation
OSDN	Open-set domain network
DoS	Denial-of-service
DDoS	Distributed denial-of-service
LightGBM	Light Gradient Boosting Machine

## Appendix A. Implementation Disclosure and Reproducibility Artefacts

This appendix records the implementation and artefact disclosures used to support the reproducibility claims made in the main paper. The disclosure is intentionally compact: it identifies the executed benchmark conditions, method roles, anchor settings, repeated run policy, leakage controls, and repository artefacts. The executable scripts, environment files, split manifests, feature admissibility records, label mapping files, prediction files, metric summaries, paired statistical outputs, and per-run evidence bundles are preserved in the accompanying repository.

**Table A1:** Implementation and reproducibility disclosure for the closed-set benchmark.

Item	Disclosure
Evaluation scope	Closed-set heterogeneous domain adaptation only. The main benchmark contexts are C1–C8, covering SS-HDA and MS-HDA under Intersection and Union representation contracts. The confirmatory second-target contexts are T1–T4, using ToN-IoT at the 1:10 labelled target ratio.
Target datasets	Edge-IIoTset is used as the main fixed IoT/IIoT target dataset for C1–C8. ToN-IoT is used as a compact second-target confirmatory dataset for T1–T4.
Source configurations	CICIDS2017, UNSW-NB15, CICIDS2017 + UNSW-NB15, and CICIDS2017 + NSL-KDD are used for the Edge-IIoTset contexts according to the context definitions in the main paper and the split summary in Table A2. CICIDS2017 and CICIDS2017 + UNSW-NB15 are used for the ToN-IoT confirmatory contexts according to Table 6.
Method roles	GGA is treated as the primary selected SS-HDA exemplar in C1–C4 and T1–T2. JSTN is treated as the primary selected MS-HDA exemplar in C5–C8 and T3–T4. PMGN and CWAN are used as 1:10 method coverage checks for C1–C4 and C5–C8, respectively, and are not pooled with the primary GGA/JSTN rows.
Representation contracts	Each context is executed under a declared representation contract. Intersection retains only admissible shared features, while Union materialises a deterministic admissible superset defined before execution. Feature admissibility decisions and excluded feature lists are stored as repository artefacts and summarised in the Appendices.
Label contracts	All reported closed-set results use the declared five-class contract: normal, scan, DoS, DDoS, and exploit/credential abuse. Raw-label mappings, merged families, and excluded target-specific families are recorded in the label mapping appendix and repository manifest.
Repeated run policy	Each fixed method–context–ratio configuration is repeated across twenty seeds: {11, 17, 23, 31, 47, 53, 59, 67, 71, 79, 83, 89, 97, 101, 103, 107, 109, 113, 127, 131}.
Paired comparison rule	For DA-versus-matched-budget target-only comparisons, paired differences are computed under the same context, representation contract, labelled target ratio, seed, and labelled target subset. The per-run split manifest records the seed-specific labelled-target subset identity used for both the DA run and the matched-budget target-only anchor.
Primary metric	Macro-F1 on the fixed target test split. Accuracy and Weighted-F1 are retained as secondary operational summaries. GapClosure, same-budget GapClosure difference, residual headroom, and paired statistical outputs are derived from the Macro-F1 records.
Neural optimiser and training budget	Adam optimiser; initial learning rate $1\times 10^{-3}$; weight decay $1\times 10^{-4}$; mini-batch size 256; maximum training budget 100 epochs; early stopping patience 15 epochs.
Model selection rule	Model selection uses only context-permitted validation data. The fixed target test split is never used for preprocessing fit, hyperparameter selection, early stopping, threshold selection, model selection, or post hoc calibration.
Anchor learner	Source-only, CORAL, matched-budget target-only, and oracle target-only anchors use the same LightGBM multiclass reference learner. Fixed settings are learning rate = 0.05, num_leaves = 31, feature_fraction = 0.8, bagging_fraction = 0.8, bagging_freq = 1, min_data_in_leaf = 20, maximum rounds = 500, and early stopping = 50 rounds.
Paired statistical outputs	For DA-versus-matched-budget target-only comparisons, the repository stores seed-level paired difference records, bootstrap confidence intervals, paired *t*-test outputs, Wilcoxon signed-rank outputs, effect-size summaries, and Holm–Bonferroni correction records. The Wilcoxon output record includes the computation mode used for each comparison, distinguishing exact signed-rank calculations from asymptotic calculations where ties or zero paired differences require a fallback. The full paired statistical outputs are reported in Appendix F.
Software and hardware manifest	The repository contains the execution environment file and hardware manifest, including the operating system, Python version 3.11.6, package versions, CPU, memory, accelerator configuration where applicable, random-seed configuration, and execution timestamp.
Repository artefacts	The repository stores executable scripts, configuration files, context specifications, split manifests, feature admissibility records, excluded feature lists, label mapping files, prediction files, score files where available, per-run metric summaries, GapClosure summaries, seed-level paired difference records, paired statistical test outputs, ToN-IoT confirmatory manifests, and evidence bundle records (schema in Appendix G).

This table reports the manuscript-level disclosure. The repository preserves the corresponding machine-readable execution records used to verify context identity, target test identity, split identity, labelled target subset identity, method configuration, anchor configuration, statistical test provenance, and output provenance.

## Appendix B. Split Manifest Summary

This appendix summarises the split cardinalities used by the closed-set benchmark. The split manifests bind each context family to its source partitions, labelled target subset, unlabelled target pool, validation split, fixed target test identity, representation contract, and seed-specific labelled-target subset identity. The tables are included to make the experimental design inspectable in the manuscript, while the repository contains the full machine-readable split manifests, per-seed labelled-subset records, and context fingerprints.

**Table A2:** Edge-IIoTset closed-set context family split constants for C1–C8.

Ctx.	Regime	Domain Tuple	Source Train	Source Val.	Target Unlabelled	Target Val.	Target Test
C1/C2	SS-HDA	CICIDS2017 → Edge-IIoTset	160,000	20,000	100,000	20,000	200,000
C3/C4	SS-HDA	UNSW-NB15 → Edge-IIoTset	140,000	18,000	100,000	20,000	200,000
C5/C6	MS-HDA	CICIDS2017 + UNSW-NB15 → Edge-IIoTset	160,000 + 140,000	20,000 + 18,000	100,000	20,000	200,000
C7/C8	MS-HDA	CICIDS2017 + NSL-KDD → Edge-IIoTset	160,000 + 18,000	20,000 + 4500	100,000	20,000	200,000

Context pairs share the same split cardinalities and differ by representation protocol: C1/C3/C5/C7 use Intersection, while C2/C4/C6/C8 use Union. For MS-HDA contexts, source cardinalities are shown as source 1 + source 2. The fixed Edge-IIoTset target unlabelled pool, target validation split, and target test split remain unchanged across labelled target ratios. The exact row identities, class stratification decisions, and context fingerprints are stored in the machine-readable split manifests.

**Table A3:** Edge-IIoTset ratio-specific labelled target schedule for C1–C8.

Ratio	Target Labelled Subset	Interpretation
1:50	2000	Severe labelled target scarcity.
1:10	10,000	Moderate labelled target budget.
1:5	20,000	Largest labelled target budget in the Edge-IIoTset closed-set benchmark.

The labelled target schedule applies to the Edge-IIoTset SS-HDA and MS-HDA context families in Table A2. The ratio changes only the labelled target subset size; it does not change the unlabelled target pool, target validation split, or fixed target test split. For paired DA-versus-matched-budget target-only testing, the same seed-specific labelled target subset is used by the DA run and the matched-budget target-only anchor.

The ToN-IoT confirmatory context definitions are listed in Table 6 in the main paper. This appendix therefore reports only the corresponding ToN-IoT split cardinalities and per-class target counts, avoiding duplication of the context definition table.

**Table A4:** ToN-IoT confirmatory target split cardinalities for T1–T4.

Target	Target Train Pool	Labelled Budget	Effective Labelled Train	Target Validation	Target Test
ToN-IoT	120,000	12,000	9600	2400	40,000

The ToN-IoT confirmatory contexts use a 10% labelled target budget from the fixed 120,000-record target training pool. This gives 12,000 labelled records and 108,000 unlabelled records before the labelled validation split. The labelled subset is then divided into 9600 effective labelled training records and 2400 validation records using the same seed-specific split rule for the DA run and the matched-budget target-only anchor. The 40,000-record target test split is fixed before execution and reflects the smaller available ToN-IoT post-preprocessing pool relative to Edge-IIoTset. *Ratio convention.* For Edge-IIoTset C1–C8, 1:10 follows the strict convention stated in the main paper: one labelled target-training instance for every ten unlabelled target-training instances. The ToN-IoT confirmatory contexts instead implement the headline scarcity level as a 10% labelled target budget from the fixed target training pool. The resulting labelled-to-unlabelled ratio before validation is 12,000:108,000=1:9. This definitional difference is disclosed here and the ToN-IoT results are interpreted only as confirmatory target sensitivity evidence, not as pooled ratio-equivalent evidence with C1–C8.

For the ToN-IoT confirmatory contexts, the target split is disclosed separately because the confirmatory experiment uses a 10% labelled target budget from a smaller fixed target-training pool rather than the Edge-IIoTset strict labelled-to-unlabelled ratio schedule. The ToN-IoT rows are therefore used to support target sensitivity analysis only and are not pooled with the Edge-IIoTset C1–C8 ratio family summaries.

**Table A5:** ToN-IoT confirmatory per-class target split counts.

Closed-Set Class	Target Train Pool	Labelled Budget	Effective Labelled Train	Target Validation	Target Test
normal	36,000	3600	2880	720	12,000
scan	24,000	2400	1920	480	8000
DoS	24,000	2400	1920	480	8000
DDoS	18,000	1800	1440	360	6000
exploit/credential abuse	18,000	1800	1440	360	6000
**Total**	**120,000**	**12,000**	**9600**	**2400**	**40,000**

The class counts reflect the ToN-IoT five-class confirmatory contract used in T1–T4. The labelled budget is stratified by class and then split into effective labelled training and validation subsets. The exact seed-specific row identities are recorded in the split manifest for reproducibility and paired statistical testing.

## Appendix C. Raw Label Mapping and Label Contract Disclosure

This appendix expands the benchmark-level label contract summary in the main text. The purpose is not to preserve each dataset’s original taxonomy but to disclose how raw families were resolved into the five-class closed-set benchmark contract:

$$Y_{\mathrm{cs}}=\left\{\mathrm{normal},\mathrm{scan},\mathrm{DoS},\mathrm{DDoS},\mathrm{exploit}/\mathrm{credential}\;\mathrm{abuse}\right\}.$$

Raw labels that could not be credibly aligned with the shared closed-set contract are excluded from this paper rather than treated as target-only unknown classes. The resulting Macro-F1, GapClosure, same-budget comparisons, and paired statistical tests are therefore conditioned on this declared five-class contract.

**Table A6:** Rationale for closed-set label contract merge and exclusion decisions.

Dataset Family	Justification
CICIDS2017	Fine-grained attack families were merged only where a broader benchmark class had a credible cross-dataset counterpart in the selected source–target tuples. Bot, Infiltration, and Heartbleed were excluded because they did not support a stable shared closed-set contract and would have introduced tuple-specific classes rather than comparable benchmark classes.
UNSW-NB15	Source-native families were consolidated into broader benchmark classes to match the benchmark’s coarse closed-set contract rather than the original dataset taxonomy. Reconnaissance is mapped to scan, DoS is retained as DoS, and Exploits are merged into exploit/credential abuse. The Generic family is mapped under the DDoS/disruptive traffic class only as a coarse flow statistics approximation: in the realised benchmark interface, it provides high-volume disruptive traffic patterns that are closer to the retained DDoS-style operational class than to the exploit/credential-abuse class, although its original semantic definition is not identical to that of DDoS. Backdoor, Shellcode, Analysis, Fuzzers, and Worms were excluded because credible one-to-one alignment with the retained benchmark classes was weak or absent across the selected tuples. Retaining them would therefore have reduced the comparability.
NSL-KDD	The legacy taxonomy was mapped to the benchmark’s coarse classes only where a stable semantic correspondence was defensible, such as Probe to scan and R2L/U2R-style behaviours to exploit/credential abuse. NSL-KDD does not provide a stable direct DDoS family under the standard taxonomy; in C7/C8, DDoS support is therefore provided by the CICIDS2017 source side where applicable. The dataset is used only as a legacy auxiliary source in MS-HDA, and its limitations are disclosed rather than hidden through artificial relabelling.
Edge-IIoTset	Target-side labels were consolidated into shared benchmark classes to create a stable closed-set evaluation target across heterogeneous sources. MITM, Backdoor, Ransomware, and Uploading were excluded because they were not credibly supported across the selected source tuples and would have made the target contract richer than the transferable source-side contract.
ToN-IoT	The confirmatory target is mapped into the same five-class closed-set contract used for the Edge-IIoTset benchmark. Normal, scan, DoS, DDoS, and exploit/credential abuse are retained where a defensible mapping exists. Injection, password, and XSS are merged into exploit/credential abuse; Backdoor, Ransomware, and MITM are excluded because they would introduce target-specific classes not stably supported by the selected source tuples.

Merge and exclusion decisions are made to preserve a stable cross-dataset benchmark contract and comparability across tuples, rather than to optimise performance within any single dataset taxonomy. The repository contains the machine-readable mapping files and spelling normalisation aliases used during preprocessing.

**Table A7:** Manuscript-level raw label mapping for the closed-set benchmark.

Benchmark Class	Raw Labels or Raw Families Mapped to the Benchmark Class	Status
**CICIDS2017**		
normal	BENIGN	Retained
scan	PortScan	Retained
DoS	DoS Hulk, DoS GoldenEye, DoS slowloris, DoS Slowhttptest	Merged
DDoS	DDoS	Retained
exploit/credential abuse	FTP-Patator, SSH-Patator, Web Attack – Brute Force, Web Attack – XSS, Web Attack – Sql Injection	Merged
**(Excluded families)**	Bot, Infiltration, Heartbleed	Excluded from closed-set contract because these families have weak or unstable correspondence to the retained five-class benchmark contract in the selected source–target tuples.
**UNSW-NB15**		
normal	Normal	Retained
scan	Reconnaissance	Merged
DoS	DoS	Retained
DDoS	Generic	Mapped under the benchmark’s coarse disruptive traffic convention; treated as a flow statistics approximation rather than a semantic DDoS label.
exploit/credential abuse	Exploits	Merged
**(Excluded families)**	Analysis, Backdoor, Fuzzers, Shellcode, Worms	Excluded from closed-set contract
**NSL-KDD**		
normal	normal	Retained
scan	ipsweep, nmap, portsweep, satan, mscan, saint	Probe family merge
DoS	back, land, neptune, pod, smurf, teardrop, apache2, mailbomb, processtable, udpstorm	Merged
DDoS	No direct stable raw DDoS family is available in the standard NSL-KDD taxonomy. In C7/C8, DDoS class supervision is therefore source-asymmetric: CICIDS2017 provides DDoS examples, while NSL-KDD does not contribute DDoS-labelled training instances. This asymmetry is preserved in the benchmark rather than addressed through synthetic relabelling.	Not mapped for NSL-KDD
exploit/credential abuse	R2L/U2R-style families, including ftp_write, guess_passwd, imap, multihop, phf, warezclient, warezmaster, buffer_overflow, loadmodule, perl, and rootkit.	Merged where retained
**(Excluded families)**	Rare or unstable raw aliases without sufficient class support after benchmark filtering, including standard NSL-KDD rare-family aliases where applicable.	Excluded from closed-set contract
**Edge-IIoTset main target**		
normal	Normal or dataset-equivalent benign label.	Retained
scan	Port_Scanning, Fingerprinting, Vulnerability_scanner	Merged
DoS	DoS_HTTP, DoS_TCP, DoS_UDP, DoS_ICMP, and spelling-normalised aliases.	Merged
DDoS	DDoS_HTTP, DDoS_TCP, DDoS_UDP, DDoS_ICMP, and spelling-normalised aliases.	Merged
exploit/credential abuse	Password, SQL_injection, XSS, and spelling-normalised aliases.	Merged
**(Excluded families)**	MITM, Backdoor, Ransomware, Uploading, and any additional raw families present in the preprocessed Edge-IIoTset variant that are not listed under the retained five-class contract.	Excluded from closed-set contract; the complete machine-readable raw label disposition list is preserved in the repository.
**ToN-IoT confirmatory target**		
normal	normal or dataset-equivalent benign label.	Retained
scan	scanning, scan, and spelling-normalised aliases.	Merged
DoS	dos and spelling-normalised aliases.	Retained
DDoS	ddos and spelling-normalised aliases.	Retained
exploit/credential abuse	injection, password, xss, and spelling-normalised aliases.	Merged
**(Excluded families)**	backdoor, ransomware, mitm	Excluded from closed-set confirmatory contract

The table reports the manuscript-level mapping used to define the five-class closed-set benchmark contract. The repository contains the exact machine-readable mapping files, spelling normalisation aliases, retained class filters, excluded family records, and class count summaries used during preprocessing. Dataset-specific raw labels are mapped only where a defensible shared benchmark class exists; unsupported families are excluded rather than treated as target-only unknown classes in this closed-set paper.

## Appendix D. Excluded-Feature Lists for Edge-IIoTset Representation Contracts

This appendix expands Table 3 by listing the features excluded during representation contract construction for the Edge-IIoTset main benchmark contexts C1–C8. The purpose is to make the feature admissibility process reproducible rather than reporting only exclusion counts. Each row records the affected context family, representation contract, dataset, or source in which the feature was observed; the original feature name; the normalised feature name where applicable; and the exclusion rationale.

The exclusion rationales distinguish between identifier-like fields, timestamp-derived fields, label-proximal fields, payload-like or free-text fields, protocol-specific metadata, source-specific legacy telemetry, endpoint metadata, and record management metadata. The exclusion lists are tuple-specific: a feature is listed only when it was present as a candidate field, dataset-specific identifier, dataset-specific timestamp, label-proximal field, or otherwise admissibility-relevant entry for that source–target combination. Features absent from a source catalogue do not appear as exclusions in tuples where they were never candidates. Union-only materialised features are not counted as excluded features; they are retained fields that are absent from at least one domain and are materialised according to the rule described in Section 3.3. The ToN-IoT confirmatory excluded feature decisions are reported separately in Appendix E.

**Table A8:** Complete excluded feature list for the Edge-IIoTset representation contracts.

Context (s)	Contract (s)	Dataset/Source	Original Feature Name	Normalised Feature Name	Exclusion Rationale
**C1/C2: CICIDS2017 → Edge-IIoTset; 11 excluded features**					
C1; C2	C1 = Intersection; C2 = Union	CICIDS2017	Flow ID	flow_id	Identifier-like field; excluded to avoid record-level artefacts and non-transferable metadata.
C1; C2	C1 = Intersection; C2 = Union	CICIDS2017	Source IP	src_ip	Identifier-like field; excluded to avoid host identity leakage and non-transferable addressing artefacts.
C1; C2	C1 = Intersection; C2 = Union	CICIDS2017	Destination IP	dst_ip	Identifier-like field; excluded to avoid host identity leakage and non-transferable addressing artefacts.
C1; C2	C1 = Intersection; C2 = Union	CICIDS2017	Timestamp	timestamp	Timestamp-derived field; excluded to avoid capture schedule artefacts and non-transferable temporal metadata.
C1; C2	C1 = Intersection; C2 = Union	CICIDS2017	Label	label	Label-proximal field; excluded because it directly encodes the prediction target.
C1; C2	C1 = Intersection; C2 = Union	Edge-IIoTset	frame.time	timestamp	Timestamp-derived field; excluded to avoid capture schedule artefacts and non-transferable temporal metadata.
C1; C2	C1 = Intersection; C2 = Union	Edge-IIoTset	ip.src_host	src_ip	Identifier-like field; excluded to avoid host identity leakage and non-transferable addressing artefacts.
C1; C2	C1 = Intersection; C2 = Union	Edge-IIoTset	ip.dst_host	dst_ip	Identifier-like field; excluded to avoid host identity leakage and non-transferable addressing artefacts.
C1; C2	C1 = Intersection; C2 = Union	Edge-IIoTset	tcp.payload	tcp_payload	Payload-like field; excluded because raw payload content is not represented consistently across domains and risks non-transferable encoding artefacts.
C1; C2	C1 = Intersection; C2 = Union	Edge-IIoTset	http.file_data	http_file_data	Free-text or payload-like field; excluded because it cannot be represented consistently across domains.
C1; C2	C1 = Intersection; C2 = Union	Edge-IIoTset	Attack_type	attack_type	Label-proximal field; excluded because it directly records the target-side attack family.
**C3/C4: UNSW-NB15 → Edge-IIoTset; 16 excluded features**					
C3; C4	C3 = Intersection; C4 = Union	UNSW-NB15	id	record_id	Record-management metadata; excluded because it identifies dataset rows rather than transferable network behaviour.
C3; C4	C3 = Intersection; C4 = Union	UNSW-NB15	srcip	src_ip	Identifier-like field; excluded to avoid host identity leakage and non-transferable addressing artefacts.
C3; C4	C3 = Intersection; C4 = Union	UNSW-NB15	dstip	dst_ip	Identifier-like field; excluded to avoid host identity leakage and non-transferable addressing artefacts.
C3; C4	C3 = Intersection; C4 = Union	UNSW-NB15	sport	src_port	Endpoint metadata; excluded specifically for the UNSW-NB15 → Edge-IIoTset tuple because the UNSW raw port representation could not be reconciled with the target-side port encoding under the declared representation contracts.
C3; C4	C3 = Intersection; C4 = Union	UNSW-NB15	dsport	dst_port	Endpoint metadata; excluded specifically for the UNSW-NB15 → Edge-IIoTset tuple because the UNSW raw port representation could not be reconciled with the target-side port encoding under the declared representation contracts.
C3; C4	C3 = Intersection; C4 = Union	UNSW-NB15	Stime	start_time	Timestamp-derived field; excluded to avoid capture schedule artefacts and non-transferable temporal metadata.
C3; C4	C3 = Intersection; C4 = Union	UNSW-NB15	Ltime	last_time	Timestamp-derived field; excluded to avoid capture schedule artefacts and non-transferable temporal metadata.
C3; C4	C3 = Intersection; C4 = Union	UNSW-NB15	attack_cat	attack_category	Label-proximal field; excluded because it records the source-side attack family.
C3; C4	C3 = Intersection; C4 = Union	UNSW-NB15	Label	label	Label-proximal field; excluded because it directly encodes the prediction target.
C3; C4	C3 = Intersection; C4 = Union	Edge-IIoTset	frame.time	timestamp	Timestamp-derived field; excluded to avoid capture schedule artefacts and non-transferable temporal metadata.
C3; C4	C3 = Intersection; C4 = Union	Edge-IIoTset	ip.src_host	src_ip	Identifier-like field; excluded to avoid host identity leakage and non-transferable addressing artefacts.
C3; C4	C3 = Intersection; C4 = Union	Edge-IIoTset	ip.dst_host	dst_ip	Identifier-like field; excluded to avoid host identity leakage and non-transferable addressing artefacts.
C3; C4	C3 = Intersection; C4 = Union	Edge-IIoTset	http.file_data	http_file_data	Free-text or payload-like field; excluded because it cannot be represented consistently across domains.
C3; C4	C3 = Intersection; C4 = Union	Edge-IIoTset	http.request.full_uri	http_request_full_uri	Free-text or unstable string field; excluded because URI strings are not consistently transferable across domains.
C3; C4	C3 = Intersection; C4 = Union	Edge-IIoTset	mqtt.topic	mqtt_topic	Protocol-specific categorical metadata; excluded because it is target-specific and not retained in the executable source–target interface.
C3; C4	C3 = Intersection; C4 = Union	Edge-IIoTset	Attack_type	attack_type	Label-proximal field; excluded because it directly records the target-side attack family.
**C5/C6: CICIDS2017 + UNSW-NB15 → Edge-IIoTset; 19 excluded features**					
C5; C6	C5 = Intersection; C6 = Union	CICIDS2017	Flow ID	flow_id	Identifier-like field; excluded to avoid record-level artefacts and non-transferable metadata.
C5; C6	C5 = Intersection; C6 = Union	CICIDS2017	Source IP	src_ip	Identifier-like field; excluded to avoid host identity leakage and non-transferable addressing artefacts.
C5; C6	C5 = Intersection; C6 = Union	CICIDS2017	Destination IP	dst_ip	Identifier-like field; excluded to avoid host identity leakage and non-transferable addressing artefacts.
C5; C6	C5 = Intersection; C6 = Union	CICIDS2017	Timestamp	timestamp	Timestamp-derived field; excluded to avoid capture schedule artefacts and non-transferable temporal metadata.
C5; C6	C5 = Intersection; C6 = Union	CICIDS2017	Label	label	Label-proximal field; excluded because it directly encodes the prediction target.
C5; C6	C5 = Intersection; C6 = Union	UNSW-NB15	id	record_id	Record-management metadata; excluded because it identifies dataset rows rather than transferable network behaviour.
C5; C6	C5 = Intersection; C6 = Union	UNSW-NB15	srcip	src_ip	Identifier-like field; excluded to avoid host identity leakage and non-transferable addressing artefacts.
C5; C6	C5 = Intersection; C6 = Union	UNSW-NB15	dstip	dst_ip	Identifier-like field; excluded to avoid host identity leakage and non-transferable addressing artefacts.
C5; C6	C5 = Intersection; C6 = Union	UNSW-NB15	Stime	start_time	Timestamp-derived field; excluded to avoid capture schedule artefacts and non-transferable temporal metadata.
C5; C6	C5 = Intersection; C6 = Union	UNSW-NB15	Ltime	last_time	Timestamp-derived field; excluded to avoid capture schedule artefacts and non-transferable temporal metadata.
C5; C6	C5 = Intersection; C6 = Union	UNSW-NB15	attack_cat	attack_category	Label-proximal field; excluded because it records the source-side attack family.
C5; C6	C5 = Intersection; C6 = Union	UNSW-NB15	Label	label	Label-proximal field; excluded because it directly encodes the prediction target.
C5; C6	C5 = Intersection; C6 = Union	Edge-IIoTset	frame.time	timestamp	Timestamp-derived field; excluded to avoid capture schedule artefacts and non-transferable temporal metadata.
C5; C6	C5 = Intersection; C6 = Union	Edge-IIoTset	ip.src_host	src_ip	Identifier-like field; excluded to avoid host identity leakage and non-transferable addressing artefacts.
C5; C6	C5 = Intersection; C6 = Union	Edge-IIoTset	ip.dst_host	dst_ip	Identifier-like field; excluded to avoid host identity leakage and non-transferable addressing artefacts.
C5; C6	C5 = Intersection; C6 = Union	Edge-IIoTset	http.file_data	http_file_data	Free-text or payload-like field; excluded because it cannot be represented consistently across domains.
C5; C6	C5 = Intersection; C6 = Union	Edge-IIoTset	http.request.full_uri	http_request_full_uri	Free-text or unstable string field; excluded because URI strings are not consistently transferable across domains.
C5; C6	C5 = Intersection; C6 = Union	Edge-IIoTset	mqtt.topic	mqtt_topic	Protocol-specific categorical metadata; excluded because it is target-specific and not retained in the executable multi-source interface.
C5; C6	C5 = Intersection; C6 = Union	Edge-IIoTset	Attack_type	attack_type	Label-proximal field; excluded because it directly records the target-side attack family.
**C7/C8: CICIDS2017 + NSL-KDD → Edge-IIoTset; 20 excluded features**					
C7; C8	C7 = Intersection; C8 = Union	CICIDS2017	Flow ID	flow_id	Identifier-like field; excluded to avoid record-level artefacts and non-transferable metadata.
C7; C8	C7 = Intersection; C8 = Union	CICIDS2017	Source IP	src_ip	Identifier-like field; excluded to avoid host identity leakage and non-transferable addressing artefacts.
C7; C8	C7 = Intersection; C8 = Union	CICIDS2017	Destination IP	dst_ip	Identifier-like field; excluded to avoid host identity leakage and non-transferable addressing artefacts.
C7; C8	C7 = Intersection; C8 = Union	CICIDS2017	Timestamp	timestamp	Timestamp-derived field; excluded to avoid capture schedule artefacts and non-transferable temporal metadata.
C7; C8	C7 = Intersection; C8 = Union	CICIDS2017	Label	label	Label-proximal field; excluded because it directly encodes the prediction target.
C7; C8	C7 = Intersection; C8 = Union	NSL-KDD	label	label	Label-proximal field; excluded because it directly encodes the prediction target.
C7; C8	C7 = Intersection; C8 = Union	NSL-KDD	difficulty	difficulty_level	Record-management metadata; excluded because it describes dataset construction rather than transferable network behaviour.
C7; C8	C7 = Intersection; C8 = Union	NSL-KDD	num_outbound_cmds	num_outbound_cmds	Source-specific legacy telemetry; excluded because it is constant or unsupported in the target feature interface.
C7; C8	C7 = Intersection; C8 = Union	NSL-KDD	is_host_login	is_host_login	Source-specific legacy telemetry; excluded because it is not retained in the target-side executable interface.
C7; C8	C7 = Intersection; C8 = Union	NSL-KDD	hot	hot	Source-only content-derived telemetry; excluded because the target does not provide a comparable measurement.
**C7/C8: CICIDS2017 + NSL-KDD → Edge-IIoTset; 20 excluded features**					
C7; C8	C7 = Intersection; C8 = Union	NSL-KDD	num_failed_logins	num_failed_logins	Source-only host/session telemetry; excluded because the target does not provide a comparable measurement.
C7; C8	C7 = Intersection; C8 = Union	NSL-KDD	num_compromised	num_compromised	Source-only host-compromise telemetry; excluded because the target does not provide a comparable measurement.
C7; C8	C7 = Intersection; C8 = Union	Edge-IIoTset	frame.time	timestamp	Timestamp-derived field; excluded to avoid capture schedule artefacts and non-transferable temporal metadata.
C7; C8	C7 = Intersection; C8 = Union	Edge-IIoTset	ip.src_host	src_ip	Identifier-like field; excluded to avoid host identity leakage and non-transferable addressing artefacts.
C7; C8	C7 = Intersection; C8 = Union	Edge-IIoTset	ip.dst_host	dst_ip	Identifier-like field; excluded to avoid host identity leakage and non-transferable addressing artefacts.
C7; C8	C7 = Intersection; C8 = Union	Edge-IIoTset	tcp.payload	tcp_payload	Payload-like field; excluded because raw payload content is not represented consistently across domains and risks non-transferable encoding artefacts.
C7; C8	C7 = Intersection; C8 = Union	Edge-IIoTset	http.file_data	http_file_data	Free-text or payload-like field; excluded because it cannot be represented consistently across domains.
C7; C8	C7 = Intersection; C8 = Union	Edge-IIoTset	http.request.full_uri	http_request_full_uri	Free-text or unstable string field; excluded because URI strings are not consistently transferable across domains.
C7; C8	C7 = Intersection; C8 = Union	Edge-IIoTset	mqtt.topic	mqtt_topic	Protocol-specific categorical metadata; excluded because it is target-specific and not retained in the executable multi-source interface.
C7; C8	C7 = Intersection; C8 = Union	Edge-IIoTset	Attack_type	attack_type	Label-proximal field; excluded because it directly records the target-side attack family.

The table reports the manuscript-level mapping used to define the five-class closed-set benchmark contract. Spelling normalisation handles minor capitalisation, whitespace, punctuation, and separator variants in raw label strings before mapping—for example, DoS_TCP versus dos_tcp or XSS versus xss. Dataset-specific raw labels are mapped only where a defensible shared benchmark class exists; unsupported families are excluded rather than treated as target-only unknown classes in this closed-set paper. Additional release-specific raw families not listed explicitly are excluded from the closed-set contract unless they appear in one of the retained rows; the repository contains the exact machine-readable mapping files, spelling normalisation aliases, retained class filters, excluded family records, and class count summaries used during preprocessing.

## Appendix E. ToN-IoT Second-Target Confirmatory Disclosure

This appendix discloses the ToN-IoT-specific information used for the second-target confirmatory experiment. The confirmatory contexts T1–T4 are not treated as a second full benchmark; they are included to test whether the qualitative headroom recovery and same-budget deployment patterns remain visible under a different IoT/IIoT target. The context definitions and split cardinalities are reported in Appendix B, and the ToN-IoT raw-label mapping is reported in Appendix C. This appendix therefore focuses on the ToN-IoT feature-space realisation and excluded feature decisions.

The ToN-IoT confirmatory experiment follows the same closed-set evaluation discipline as for the Edge-IIoTset benchmark. Each confirmatory context declares a source–target tuple, representation contract, fixed target test identity, labelled target subset, unlabelled target-training pool, anchor ladder, and method role before execution. The four confirmatory contexts are CICIDS2017 → ToN-IoT under Intersection and Union for SS-HDA, and CICIDS2017 + UNSW-NB15 → ToN-IoT under Intersection and Union for MS-HDA. The same twenty-seed repeated run policy and paired DA-versus-matched-budget target-only comparison rule are used.

**Table A9:** ToN-IoT second-target confirmatory feature-space summary.

Ctx.	Domain Tuple	Protocol	Shared Raw Candidates	Retained Features	Union-Only Materialised	Excluded
T1	CICIDS2017 → ToN-IoT	Intersection	36	24	0	12
T2	CICIDS2017 → ToN-IoT	Union	36	43	19	12
T3	CICIDS2017 + UNSW-NB15 → ToN-IoT	Intersection	25	21	0	17
T4	CICIDS2017 + UNSW-NB15 → ToN-IoT	Union	25	52	31	17

Shared raw candidates are the admissible source–target feature concepts available before final representation contract realisation. Retained features are the final executable feature concepts under the declared representation contract. Union-only materialised features are retained fields that are absent from at least one domain and are deterministically materialised under the Union contract. They are not counted as exclusions. The “Excluded” column counts feature entries excluded across the source and target datasets, including identifier-like, timestamp-derived, label-proximal, endpoint, and record management fields. Excluded entries may exceed the difference between shared raw candidates and retained features because some excluded entries are dataset-specific and were never shared raw candidates. The smaller shared-candidate count for T3/T4 reflects the stricter three-domain reconciliation required by the multi-source tuple.

**Table A10:** Complete excluded feature list for the ToN-IoT confirmatory representation contracts.

Context (s)	Contract (s)	Dataset/Source	Original Feature Name	Normalised Feature Name	Exclusion Rationale
**T1/T2: CICIDS2017 → ToN-IoT; 12 excluded features**					
T1; T2	T1 = Intersection; T2 = Union	CICIDS2017	Flow ID	flow_id	Identifier-like field; excluded to avoid record-level artefacts and non-transferable metadata.
T1; T2	T1 = Intersection; T2 = Union	CICIDS2017	Source IP	src_ip	Identifier-like field; excluded to avoid host identity leakage and non-transferable addressing artefacts.
T1; T2	T1 = Intersection; T2 = Union	CICIDS2017	Destination IP	dst_ip	Identifier-like field; excluded to avoid host identity leakage and non-transferable addressing artefacts.
T1; T2	T1 = Intersection; T2 = Union	CICIDS2017	Timestamp	timestamp	Timestamp-derived field; excluded to avoid capture schedule artefacts and non-transferable temporal metadata.
T1; T2	T1 = Intersection; T2 = Union	CICIDS2017	Label	label	Label-proximal field; excluded because it directly encodes the prediction target.
T1; T2	T1 = Intersection; T2 = Union	ToN-IoT	ts	timestamp	Timestamp-derived field; excluded to avoid capture schedule artefacts and non-transferable temporal metadata.
T1; T2	T1 = Intersection; T2 = Union	ToN-IoT	src_ip	src_ip	Identifier-like field; excluded to avoid host identity leakage and non-transferable addressing artefacts.
T1; T2	T1 = Intersection; T2 = Union	ToN-IoT	dst_ip	dst_ip	Identifier-like field; excluded to avoid host identity leakage and non-transferable addressing artefacts.
T1; T2	T1 = Intersection; T2 = Union	ToN-IoT	src_port	src_port	Endpoint metadata; excluded in the ToN-IoT confirmatory interface because the raw port representation was not retained after source–target feature reconciliation under the declared representation contracts.
T1; T2	T1 = Intersection; T2 = Union	ToN-IoT	dst_port	dst_port	Endpoint metadata; excluded in the ToN-IoT confirmatory interface because the raw port representation was not retained after source–target feature reconciliation under the declared representation contracts.
T1; T2	T1 = Intersection; T2 = Union	ToN-IoT	type	attack_type	Label-proximal field; excluded because it records the target-side attack family.
T1; T2	T1 = Intersection; T2 = Union	ToN-IoT	label	label	Label-proximal field; excluded because it directly encodes the prediction target.
**T3/T4: CICIDS2017 + UNSW-NB15 → ToN-IoT; 17 excluded features**					
T3; T4	T3 = Intersection; T4 = Union	CICIDS2017	Flow ID	flow_id	Identifier-like field; excluded to avoid record-level artefacts and non-transferable metadata.
T3; T4	T3 = Intersection; T4 = Union	CICIDS2017	Source IP	src_ip	Identifier-like field; excluded to avoid host identity leakage and non-transferable addressing artefacts.
T3; T4	T3 = Intersection; T4 = Union	CICIDS2017	Destination IP	dst_ip	Identifier-like field; excluded to avoid host identity leakage and non-transferable addressing artefacts.
T3; T4	T3 = Intersection; T4 = Union	CICIDS2017	Timestamp	timestamp	Timestamp-derived field; excluded to avoid capture schedule artefacts and non-transferable temporal metadata.
T3; T4	T3 = Intersection; T4 = Union	CICIDS2017	Label	label	Label-proximal field; excluded because it directly encodes the prediction target.
T3; T4	T3 = Intersection; T4 = Union	UNSW-NB15	id	record_id	Record-management metadata; excluded because it identifies dataset rows rather than transferable network behaviour.
T3; T4	T3 = Intersection; T4 = Union	UNSW-NB15	srcip	src_ip	Identifier-like field; excluded to avoid host identity leakage and non-transferable addressing artefacts.
T3; T4	T3 = Intersection; T4 = Union	UNSW-NB15	dstip	dst_ip	Identifier-like field; excluded to avoid host identity leakage and non-transferable addressing artefacts.
T3; T4	T3 = Intersection; T4 = Union	UNSW-NB15	Stime	start_time	Timestamp-derived field; excluded to avoid capture schedule artefacts and non-transferable temporal metadata.
T3; T4	T3 = Intersection; T4 = Union	UNSW-NB15	Ltime	last_time	Timestamp-derived field; excluded to avoid capture schedule artefacts and non-transferable temporal metadata.
T3; T4	T3 = Intersection; T4 = Union	UNSW-NB15	attack_cat	attack_category	Label-proximal field; excluded because it records the source-side attack family.
T3; T4	T3 = Intersection; T4 = Union	UNSW-NB15	Label	label	Label-proximal field; excluded because it directly encodes the prediction target.
T3; T4	T3 = Intersection; T4 = Union	ToN-IoT	ts	timestamp	Timestamp-derived field; excluded to avoid capture schedule artefacts and non-transferable temporal metadata.
T3; T4	T3 = Intersection; T4 = Union	ToN-IoT	src_ip	src_ip	Identifier-like field; excluded to avoid host identity leakage and non-transferable addressing artefacts.
T3; T4	T3 = Intersection; T4 = Union	ToN-IoT	dst_ip	dst_ip	Identifier-like field; excluded to avoid host identity leakage and non-transferable addressing artefacts.
T3; T4	T3 = Intersection; T4 = Union	ToN-IoT	type	attack_type	Label-proximal field; excluded because it records the target-side attack family.
T3; T4	T3 = Intersection; T4 = Union	ToN-IoT	label	label	Label-proximal field; excluded because it directly encodes the prediction target.

The excluded feature counts in this appendix correspond to the “Excluded” column in Table 3. Because the exclusion count is identical for each paired Intersection/Union tuple in Table 3, each source–target tuple is reported once and linked to both contexts. The “Excluded” count is an exclusion entry count across the datasets in a tuple, not simply the arithmetic difference between shared raw candidates and retained final features. Union-only materialised features are not listed as exclusions because they remain part of the realised Union feature interface. The repository contains the corresponding machine-readable feature admissibility records, including original feature names, normalised names, exclusion rationales, and representation contract hashes.

## Appendix F. Paired Statistical Test Outputs

This appendix reports the full paired DA-versus-matched-budget target-only statistical outputs used to support the compact statistical table in the main text. For each comparison, the paired difference is computed as the DA Macro-F1 minus the matched-budget target-only Macro-F1 under the same context, representation contract, labelled target ratio, seed, and labelled target subset. All comparisons use n=20 paired seeds.

Hypothesis tests are corrected within three declared inference families. The first family contains the 24 primary Edge-IIoTset GGA/JSTN comparisons across C1–C8 and the three labelled target ratios. The second family contains the eight 1:10 PMGN/CWAN method coverage comparisons on Edge-IIoTset. The third family contains the four ToN-IoT 1:10 confirmatory comparisons. This separation follows the research question structure of the paper: primary deployment comparison, method selection sensitivity, and target dependence confirmation are distinct inferential questions. Table A11 reports all 36 paired comparisons across these three families: 24 primary, 8 method coverage, and 4 confirmatory.

**Table A11:** Full paired statistical outputs for DA versus matched-budget target-only learning.

Ctx.	Method	Ratio	Protocol	Mean Paired ΔF1	95% CI	Adj. $p_{t}$	Adj. $p_{W}$	$d_{z}$	$r_{\mathrm{rb}}$
**Primary Edge-IIoTset comparisons: GGA/JSTN, C1–C8, all ratios**									
C1	GGA	1:50	Intersection	+0.026	[0.024,0.028]	<0.001	<0.001	5.427	1.000
C1	GGA	1:10	Intersection	−0.001	[−0.003,0.002]	0.601	0.240	−0.119	−0.368
C1	GGA	1:5	Intersection	−0.016	[−0.018,−0.013]	<0.001	<0.001	−2.903	−1.000
C2	GGA	1:50	Union	−0.014	[−0.016,−0.012]	<0.001	<0.001	−3.063	−1.000
C2	GGA	1:10	Union	−0.018	[−0.020,−0.016]	<0.001	<0.001	−3.838	−1.000
C2	GGA	1:5	Union	−0.038	[−0.040,−0.036]	<0.001	<0.001	−7.110	−1.000
C3	GGA	1:50	Intersection	−0.019	[−0.022,−0.017]	<0.001	<0.001	−3.204	−1.000
C3	GGA	1:10	Intersection	−0.032	[−0.034,−0.029]	<0.001	<0.001	−6.351	−1.000
C3	GGA	1:5	Intersection	−0.050	[−0.053,−0.047]	<0.001	<0.001	−6.749	−1.000
C4	GGA	1:50	Union	−0.047	[−0.049,−0.045]	<0.001	<0.001	−9.223	−1.000
C4	GGA	1:10	Union	−0.070	[−0.073,−0.068]	<0.001	<0.001	−12.066	−1.000
C4	GGA	1:5	Union	−0.082	[−0.085,−0.080]	<0.001	<0.001	−14.971	−1.000
C5	JSTN	1:50	Intersection	−0.017	[−0.019,−0.015]	<0.001	<0.001	−3.428	−1.000
C5	JSTN	1:10	Intersection	−0.050	[−0.052,−0.049]	<0.001	<0.001	−14.843	−1.000
C5	JSTN	1:5	Intersection	−0.049	[−0.051,−0.047]	<0.001	<0.001	−10.103	−1.000
C6	JSTN	1:50	Union	+0.023	[0.020,0.025]	<0.001	<0.001	4.260	1.000
C6	JSTN	1:10	Union	−0.002	[−0.004,0.000]	0.272	0.240	−0.348	−0.444
C6	JSTN	1:5	Union	−0.011	[−0.013,−0.008]	<0.001	<0.001	−1.901	−1.000
C7	JSTN	1:50	Intersection	+0.027	[0.024,0.029]	<0.001	<0.001	3.935	1.000
C7	JSTN	1:10	Intersection	+0.025	[0.023,0.028]	<0.001	<0.001	4.388	1.000
C7	JSTN	1:5	Intersection	+0.006	[0.004,0.008]	<0.001	<0.001	1.402	0.962
C8	JSTN	1:50	Union	−0.006	[−0.008,−0.003]	0.001	0.001	−0.958	−0.868
C8	JSTN	1:10	Union	−0.031	[−0.033,−0.028]	<0.001	<0.001	−5.663	−1.000
C8	JSTN	1:5	Union	−0.030	[−0.032,−0.027]	<0.001	<0.001	−4.452	−1.000
**Edge-IIoTset method coverage comparisons: PMGN/CWAN at 1:10**									
C1	PMGN	1:10	Intersection	−0.006	[−0.008,−0.004]	<0.001	<0.001	−1.122	−0.980
C2	PMGN	1:10	Union	−0.026	[−0.028,−0.024]	<0.001	<0.001	−6.007	−1.000
C3	PMGN	1:10	Intersection	−0.041	[−0.044,−0.038]	<0.001	<0.001	−6.451	−1.000
C4	PMGN	1:10	Union	−0.083	[−0.086,−0.080]	<0.001	<0.001	−10.366	−1.000
C5	CWAN	1:10	Intersection	−0.036	[−0.038,−0.034]	<0.001	<0.001	−7.360	−1.000
C6	CWAN	1:10	Union	+0.010	[0.007,0.013]	<0.001	<0.001	1.359	0.929
C7	CWAN	1:10	Intersection	+0.018	[0.016,0.020]	<0.001	<0.001	4.212	1.000
C8	CWAN	1:10	Union	−0.038	[−0.041,−0.036]	<0.001	<0.001	−6.561	−1.000
**ToN-IoT second-target confirmatory comparisons at 1:10**									
T1	GGA	1:10	Intersection	+0.013	[0.011,0.015]	<0.001	<0.001	2.423	1.000
T2	GGA	1:10	Union	−0.012	[−0.015,−0.009]	<0.001	<0.001	−1.648	−0.971
T3	JSTN	1:10	Intersection	+0.019	[0.016,0.022]	<0.001	<0.001	2.805	1.000
T4	JSTN	1:10	Union	−0.005	[−0.008,−0.002]	0.006	0.007	−0.698	−0.667

ΔF1 is DA Macro-F1 minus matched-budget target-only Macro-F1 under the same seed and labelled target subset. *pt* is the paired *t*-test *p*-value and *p_W_* is theWilcoxon signed-rank *p*-value; both are Holm–Bonferroni-adjusted within the declared inference family: primary Edge-IIoTset comparisons, *n* = 24; method coverage comparisons, *n* = 8; and ToN-IoT confirmatory comparisons, *n* = 4. *d_z_* is Cohen’s paired-difference effect size, computed as $\bar{d}$/*s_d_*, and *r_rb_* is the matched-pairs rank-biserial correlation. Confidence intervals are percentile bootstrap 95% intervals for the mean paired difference based on *n* = 20 paired seeds and 10,000 bootstrap resamples.

## Appendix G. Evidence Bundle Schema

The benchmark stores one evidence bundle for each executed run. The purpose of the evidence bundle is to bind each reported score to a fixed context, split manifest, target test identity, representation contract, label contract, seed, method role, prediction file, metric record, and statistical provenance where applicable. This prevents a result from being interpreted as a method property that is independent of the benchmark context in which it was produced. The same schema is used for primary DA runs, PMGN/CWAN method coverage checks, ToN-IoT confirmatory runs, and anchor runs, with fields marked as conditional where they apply only to a subset of run types.

**Table A12:** Standardised evidence bundle schema for closed-set DA, method coverage, confirmatory, and anchor runs.

Artifact Field	Description	DA or Coverage	Anchor Runs
run_signature	Stable identifier binding method or anchor type, target dataset, context, protocol, ratio, seed, and reporting view.	Yes	Yes
target_dataset_role	Indicator of whether the run belongs to the main Edge-IIoTset benchmark target or the ToN-IoT second-target confirmatory setting.	Yes	Yes
resolved_context_spec	Fully resolved context specification, including domain tuple, supervision regime, representation contract, label contract, labelled target budget, and fixed target test identity.	Yes	Yes
context_fingerprint	Stable fingerprint or equivalent identifier for the resolved context, used to verify context identity across runs and derived analyses.	Yes	Yes
split_manifest_ref	Manifest filename or identifier, together with the split-identity record used to bind source partitions, target-training partitions, validation data, and fixed target test rows.	Yes	Yes
labelled_target_subset_ref	Seed-specific labelled-target subset identifier or hash. Required for semi-supervised DA, method coverage checks, matched-budget target-only anchors, and paired DA-versus-target-only comparisons.	Yes	Cond.
seed	Fixed random seed used for the execution.	Yes	Yes
label_contract_ref	Reference to the closed-set five-class label contract and raw-label mapping used by the run.	Yes	Yes
representation_provenance	Realised feature interface, retained columns, materialised Union-only fields where applicable, excluded feature record, preprocessing scope, applied transform sequence, and fitted transform state where applicable, such as scaler parameters or encoder vocabularies fitted only on permitted training partitions.	Yes	Yes
method_role	Declared role of the evaluated method: primary selected exemplar, method coverage check, confirmatory target run, source-only anchor, CORAL anchor, matched-budget target-only anchor, or oracle target-only anchor.	Yes	Yes
method_provenance	DA method name, declared native regime, implementation provenance, target label use contract, and fixed method configuration.	Yes	No
anchor_provenance	Anchor type, learner configuration, permitted supervision, ratio dependence flag, and target label budget where applicable.	No	Yes
train_config	Learner configuration, optimiser or learner settings, early stopping policy, fixed parameters, validation rule, and training budget record.	Yes	Yes
validation_record	Validation metric history or selected validation score used for model selection where applicable. The fixed target test split is never used for model selection.	Yes	Yes
prediction_file_ref	Path or reference to predictions on the fixed target test split.	Yes	Yes
score_file_ref	Path or reference to class scores or probabilities, where available and semantically meaningful.	Yes	Yes
metrics_summary	Target test metrics for the run, including Macro-F1, accuracy, Weighted-F1, and other reported metrics where semantically appropriate.	Yes	Yes
gapclosure_inputs	Source-only and oracle target-only references used when a run contributes to GapClosure, same-budget GapClosure difference, or residual headroom reporting.	Yes	Cond.
paired_comparison_ref	Reference to the paired DA-versus-matched-budget target-only comparison record when the run contributes to paired statistical testing.	Cond.	Cond.
statistical_family_ref	Declared inference-family identifier used for Holm–Bonferroni correction: primary Edge-IIoTset comparisons, PMGN/CWAN method coverage comparisons, or ToN-IoT confirmatory comparisons.	Cond.	Cond.
qa_core_status	PASS, WARN, or FAIL checks for split identity, target test isolation, labelled subset identity, label consistency, representation contract match, leakage boundaries, and required output completeness.	Yes	Yes
qa_extended_warnings	Scarcity warnings, class support warnings, representation sensitivity warnings, target-confirmatory warnings, and method role warnings, such as attempting to interpret a coverage check row as a primary method row or pooling confirmatory target rows with main benchmark rows.	Yes	Yes
run_status	Completion status, failure code if applicable, aggregation eligibility, and reason for exclusion from aggregation where applicable.	Yes	Yes

“Cond.” means conditional. labelled_target_subset_ref is required whenever a run uses or is paired against a seed-specific labelled target subset. gapclosure_inputs are required for DA rows, method coverage rows, ToN-IoT confirmatory rows, and matched-budget target-only rows when they are used in GapClosure-derived reporting. Source-only and oracle target-only anchors define the denominator of the GapClosure calculation and are therefore referenced by derived analyses rather than treated as ordinary DA rows. This closed-set paper does not use benchmark-side adapter-based portability rows for its primary claims. PMGN and CWAN are treated as method coverage checks under declared closed-set contexts, not as adapter-mediated portability rows. Adapter provenance fields are therefore not required for the reported closed-set primary, coverage, confirmatory, or anchor runs.

## References

[1] da Costa K.A.P., Papa J.P., Lisboa C.O., Muñoz R., de Albuquerque V.H.C. (2019). Internet of Things: A survey on machine learning-based intrusion detection approaches. Comput. Netw..

[2] Layeghy S., Baktashmotlagh M., Portmann M. (2023). DI-NIDS: Domain invariant network intrusion detection system. Knowl.-Based Syst..

[3] Yuan X., Han S., Huang W., Ye H., Kong X., Zhang F. (2024). A simple framework to enhance the adversarial robustness of deep learning-based intrusion detection system. Comput. Secur..

[4] Zhang J., Li Y., Zhang L. (2025). Heterogeneous network intrusion detection via domain adaptation in IoT environment. Internet Technol. Lett..

[5] Jin H. (2026). Cross-Protocol Domain Gap in Internet of Things Intrusion and Anomaly Detection: An Empirical Internet Protocol-to-Bluetooth Low Energy Study of Domain-Adversarial Training. Sensors.

[6] Apruzzese G., Pajola L., Conti M. (2022). The Cross-evaluation of Machine Learning-based Network Intrusion Detection Systems. IEEE Trans. Netw. Serv. Manag..

[7] Tavallaee M., Stakhanova N., Ghorbani A.A. (2010). Toward credible evaluation of anomaly-based intrusion-detection methods. IEEE Trans. Syst. Man Cybern. Part C Appl. Rev..

[8] Hamouda D., Ferrag M.A., Benhamida N., Seridi H., Ghanem M.C. (2024). Revolutionizing intrusion detection in industrial IoT with distributed learning and deep generative techniques. Internet Things.

[9] Ferrag M.A., Friha O., Kantarci B., Tihanyi N., Cordeiro L., Debbah M., Hamouda D., Al-Hawawreh M., Choo K.K.R. (2023). Edge Learning for 6G-Enabled Internet of Things: A Comprehensive Survey of Vulnerabilities, Datasets, and Defenses. IEEE Commun. Surv. Tutor..

[10] Rahman M.M., Shakil S.A., Mustakim M.R. (2025). A survey on intrusion detection system in IoT networks. Cyber Secur. Appl..

[11] Sun S., Zhou L., Wang Z., Han L. (2025). Robust intrusion detection based on personalized federated learning for IoT environment. Comput. Secur..

[12] Sommer R., Paxson V. (2010). Outside the closed world: On using machine learning for network intrusion detection. Proceedings of the 2010 IEEE Symposium on Security and Privacy.

[13] Ring M., Wunderlich S., Scheuring D., Landes D., Hotho A. (2019). A survey of network-based intrusion detection data sets. Comput. Secur..

[14] Chou D., Jiang M. (2021). A Survey on Data-driven Network Intrusion Detection. ACM Comput. Surv..

[15] Wu J., Wang Y. (2026). TriHID: Towards verifiable domain adaptation-based IoT intrusion detection in heterogeneous environment. Expert Syst. Appl..

[16] Wu J., Dai H., Wang Y., Ye K., Xu C. (2023). Heterogeneous Domain Adaptation for IoT Intrusion Detection: A Geometric Graph Alignment Approach. IEEE Internet Things J..

[17] Wu J., Wang Y., Xie B., Li S., Dai H., Ye K., Xu C. (2023). Joint Semantic Transfer Network for IoT Intrusion Detection. IEEE Internet Things J..

[18] Wang Z., Luo Y., Huang Z., Baktashmotlagh M. (2020). Prototype-Matching Graph Network for Heterogeneous Domain Adaptation. Proceedings of the 28th ACM International Conference on Multimedia (ACM MM ’20).

[19] Yao Y., Li X., Zhang Y., Ye Y. (2023). Multisource Heterogeneous Domain Adaptation With Conditional Weighting Adversarial Network. IEEE Trans. Neural Netw. Learn. Syst..

[20] Sun B., Feng J., Saenko K. (2016). Return of Frustratingly Easy Domain Adaptation. Proceedings of the AAAI Conference on Artificial Intelligence.

[21] Alsaedi A., Moustafa N., Tari Z., Mahmood A., Anwar A.N. (2020). TON-IoT telemetry dataset: A new generation dataset of IoT and IIoT for data-driven intrusion detection systems. IEEE Access.

[22] Ferrag M.A., Friha O., Hamouda D., Maglaras L., Janicke H. (2022). Edge-IIoTset: A New Comprehensive Realistic Cyber Security Dataset of IoT and IIoT Applications for Centralized and Federated Learning. IEEE Access.

[23] Wang Q., Wang X., Liu H., Wang Y., Ren J., Zhang B. (2024). A Domain Adaptive IoT Intrusion Detection Algorithm Based on GWR-GCN Feature Extraction and Conditional Domain Adversary. IEEE Internet Things J..

[24] Wu J., Dai H., Kent K.B., Yen J., Xu C., Wang Y. (2024). Open set dandelion network for IoT intrusion detection. ACM Trans. Internet Technol..

[25] Jing T., Xia H., Liu H., Ding Z. (2025). Interpretable Novel Target Discovery through Open-Set Domain Adaptation. ACM Trans. Multimed. Comput. Commun. Appl..

[26] Sharafaldin I., Lashkari A.H., Ghorbani A.A. (2018). Toward generating a new intrusion detection dataset and intrusion traffic characterization. Proceedings of the ICISSP 2018—Proceedings of the 4th International Conference on Information Systems Security and Privacy.

[27] Moustafa N., Slay J. (2017). The significant features of the UNSW-NB15 and the KDD99 data sets for Network Intrusion Detection Systems. Proceedings of the 2015 4th International Workshop on Building Analysis Datasets and Gathering Experience Returns for Security, BADGERS 2015.

[28] Ragab M., Eldele E., Tan W.L., Foo C.S., Chen Z., Wu M., Kwoh C.K., Li X. (2023). ADATIME: A Benchmarking Suite for Domain Adaptation on Time Series Data. ACM Trans. Knowl. Discov. Data.

[29] Zhao H., Zhang S., Wu G., Moura J.M.F., Costeira J.P., Gordon G.J. Adversarial Multiple Source Domain Adaptation. Proceedings of the Advances in Neural Information Processing Systems.

[30] Sokolova M., Lapalme G. (2009). A systematic analysis of performance measures for classification tasks. Inf. Process. Manag..

[31] Fawcett T. (2006). An introduction to ROC analysis. Pattern Recognit. Lett..

[32] Davis J., Goadrich M. The Relationship Between Precision-Recall and ROC Curves. Proceedings of the 23rd International Conference on Machine Learning.

[33] Kingma D.P., Ba J. Adam: A Method for Stochastic Optimization. Proceedings of the International Conference on Learning Representations (ICLR).

[34] Ke G., Meng Q., Finley T., Wang T., Chen W., Ma W., Ye Q., Liu T. LightGBM: A Highly Efficient Gradient Boosting Decision Tree. Proceedings of the Advances in Neural Information Processing Systems.

